# Youth with severe mental illness and complex non-somatic motor abnormalities: conflicting conceptualizations and unequal treatment

**DOI:** 10.1038/s44184-022-00013-8

**Published:** 2022-10-05

**Authors:** Peter Andersson, Lee E. Wachtel, Johan Lundberg, Esmail Jamshidi, Johan Bring, Mathias Rask-Andersen, Håkan Jarbin, Jussi Jokinen, Adrian E. Desai Boström

**Affiliations:** 1 Department of Clinical Neuroscience/Psychology, Karolinska Institute, Stockholm, Sweden; 2 Centre for Clinical Research Dalarna, Uppsala University, Falun, Sweden; 3 Kennedy Krieger Institute, Johns Hopkins School of Medicine, Baltimore, MD USA; 4 Centre for Psychiatry Research, Department of Clinical Neuroscience, Karolinska Institutet, & Stockholm Health Care Services, Region Stockholm, Karolinska University Hospital, SE-171 76 Stockholm, Sweden; 5 Stockholm Health Care Services, Region Stockholm, Stockholm, Sweden; 6 Department of Clinical Sciences/Psychiatry, Umeå University, Umeå, Sweden; 7 Statisticon AB, Uppsala, Sweden; 8 Department of Immunology, Genetics and Pathology, Science for Life Laboratory, Uppsala University, Uppsala, Sweden; 9 Department of Clinical Sciences Lund, Section of Child and Adolescent Psychiatry, Lund University, Lund, Sweden; 10 Child and Adolescent Psychiatry, Region Halland, Halmstad, Sweden; 11 Department of Women’s and Children’s Health/Neuropediatrics, Karolinska Institutet, Stockholm, Sweden

**Keywords:** Psychiatric disorders, Schizophrenia, Paediatric research, Diagnosis

## Abstract

Two emerging diagnostic concepts promote distinct treatments for youth with acute-onset motor abnormalities and severe concurrent psychiatric symptoms: Pediatric acute-onset neuropsychiatric syndrome (PANS) and pediatric catatonia. Both have institutional approval in parts of Europe and in the USA, meriting an unconditional comparison of supporting evidence. Here we report results of qualitative and quantitative analyses of literature and Swedish National Registry Data suggesting that (1) catatonic patients are liable to fulfilling diagnostic criteria for PANS, (2) three conservatively assessed PANS case-reports present with possible unrecognized catatonia, (3) lithium and electroconvulsive therapy usage frequencies in Swedish minors (exclusively recommended for severe mental illness) are strongly intercorrelated and unequally distributed across Swedish counties, (4) established severe mental disorders are rarely overtly considered amongst PANS-specific research and (5) best-available evidence treatments appear markedly superior for pediatric catatonia compared to PANS in both childhood and adolescence. Prioritizing treatments for pediatric catatonia in concerned subjects could markedly improve treatment outcomes.

## Introduction

Pediatric Acute-onset Neuropsychiatric Syndrome (PANS)—a descriptive symptom-based diagnosis including psychiatric and somatic symptoms with acute onset—has emerged as a diagnostic concept with growing international traction. PANS-dedicated clinics have gained institutional approval (e.g., integration of research treatment clinics into clinical practice, official media dissemination of the PANS concept) in parts of Europe^1,2^ and in the USA^3^. In 2012, Swedo et al. presented a proposition for diagnostic criteria: Abrupt-onset of obsessive-compulsive disorder or severely restricted food intake with the concurrent acute and severe presentation of at least two of the following: anxiety; emotional lability and/or depression; irritability, aggression, and/or severely oppositional behaviors; behavioral (developmental regression); deterioration in school performance; sensory or motor abnormalities and somatic symptoms including sleep disturbances, enuresis or urinary frequency^4^. In 2021, Pfeiffer et al. presented a similar proposition for consensus diagnostic criteria, specifying instead a 72-hour-period for the definition of ‘acute onset’^5^, otherwise largely comparable to items described by that of Swedo et al.^4^. In both propositions, known neurologic or medical disorders that better explain symptoms preclude a diagnosis of PANS^4,5^. The proposed criteria for PANS, however, do not explicitly state that the co-occurrence of another established mental disorder that better explains the symptoms precludes a PANS diagnosis^4,5^. The underlying pathophysiology was proposed to be related to neuroinflammatory alterations^4,5^. However, there are as of yet no objective findings implicating causality and no reliable biomarkers to guide clinicians^6^. In 2021, Johnson et al. conducted a systematic review of treatments related to PANS, concluding that treatments for PANS entail low certainty evidence of beneficial effects, and moderate certainty evidence of adverse effects^7^. Indeed, some proposed treatments such as plasma exchange therapy and intravenous immunoglobins carry substantial risks e.g., thrombosis, anaphylaxis, autonomic instability^8^, hemolysis, kidney injury, and hypersensitivity reactions^9^. The long-term prognosis of 34 PANS patients treated at a dedicated clinic has been previously described—by a median follow-up of 3.3 years, ~6% were in remission, ~59% relapse-remitting, and 35% chronic-state/progressive course^6^. The authors noted that the chronic course group was significantly more impaired at baseline—suggesting this is a useful predictor of chronicity in the context of PANS^6^.

Chronicity also exists in pediatric catatonia, a potentially life-threatening condition entailing acute onset motor, vocal and behavioral abnormalities—associated with a 60-fold increased risk of premature mortality^10^. In the DSM-5, catatonia is defined as three or more of the following symptoms: catalepsy, waxy flexibility, stupor, agitation, mutism, negativism, posturing, mannerism, stereotypies, grimacing, echolalia or echopraxia^11^. Symptom onset can be rapid or gradually increasing and the condition can be transitory or chronically present for weeks to several months. Three subtypes of catatonia are commonly described in the clinical literature; (1) hypokinetic or stuporous catatonia (perhaps the most well-known form), characterized by mutism, immobility, negativism, rigidity, posturing, and catalepsy; (2) hyperkinetic or excitatory/agitated catatonia associated with impulsivity, aggressive and excitatory behavior, and the potentially life-threatening condition (3) malignant catatonia, characterized by fever and autonomic instability^12^. While catatonic subtypes have not been explicitly recognized in the DSM-5 or ICD-11, it should be noted that both classification systems include a set of diagnostic items that allow for the diagnosis of all subtypes under the same umbrella—thus, implicitly accounting for catatonic subtypes by allowing classification of the diagnosis of catatonia without the presence of stuporous features. Regardless of subtype, previous research establish benzodiazepines and ECT as effective treatments for catatonic patients (the latter treatment generally reserved for benzodiazepine-resistant cases or subjects with life-threatening features)^13^. Specific symptoms may be more pronounced in pediatric populations, e.g., refusal to eat and drink, social withdrawal, repetitive movements, and regressive symptoms such as enuresis^14^. The condition is implicated in schizophrenia, affective disorders and has been increasingly recognized in autism spectrum disorders. Other developmental conditions, including Prader-Willis syndrome, have also been associated with pediatric catatonia^10^. Approximately 20% of reported cases of pediatric catatonia are caused by organic illness^10^. 17% of a pediatric inpatient sample satisfied stringent criteria for catatonia whereas only ~1–2% received appropriate diagnosis in a retrospective chart review^15^. Afflicted patients can progress to treatment-refractory states and chronic severe debilitation if adequate treatment is withheld^16^. Lorazepam conferred vast benefits in 65% of 66 cases studied in a prospective observational study in a pediatric population^17^. Reviews of case-reports and observational studies demonstrate robust treatment outcomes when ECT is implemented in the treatment of pediatric catatonia in 76–92% of cases studied^18^. Several retrospective studies, case-studies, and literature reviews have consistently demonstrated adequate safety profiles and robust improvements in catatonic symptoms when ECT is implemented in the acute and maintenance treatment of pediatric catatonia—with and without comorbid developmental disorders^18^. However, evidence-based conclusions regarding efficacy are prevented by the lack of comparative and/or placebo-controlled studies^19^. Today, there are no published randomized controlled trials (RCTs) of ECT as a treatment for any severe mental disorder in pediatric populations. Fewer than 20 minors in Sweden received ECT treatment in 2020^20^, encompassing a population exceeding 10 million people. This should be considered in light of national treatment guidelines (congruent with the 2004 AACAP Treatment consensus on ECT^18^)—since 2016 and with the highest priority recommending its use in the care of post-pubertal adolescents with severe major depressive disorder with mood-congruent psychotic symptoms, catatonia or treatment resistance^21^.

Recent case-studies preferentially implement immunomodulatory regimens in subjects with comorbid PANS and catatonia, deselecting recommended therapies for catatonia^22,23^. Such ambiguity arguably creates dubiety as to which treatment regimen to prioritize and as to whether severe mental conditions—such as catatonia, schizophrenia, severe major depressive disorder, or bipolar disorder—constitute exclusion criteria for PANS, if perceived to better explain the symptoms presented and not caused by a medical condition. Motor abnormalities of involuntary nature represent a key concept of catatonia^16^. In the case of PANS, their precise nature is not overtly specified, thus potentially encompassing both goal-orientated and involuntary motor symptoms. Previous studies have implicated that involuntary motor abnormalities are often overlooked in clinical practice and misattributed to other conditions, contributing to the putative under recognition of the catatonic syndrome^16^. For example, several adolescents and young adults with tics were shown to fulfill the criteria for pediatric catatonia, and adolescents with co-occurring obsessive–compulsive disorder (OCD) presented with co-morbid catatonia—all markedly improved by Lorazepam or ECT treatment^24,25^. Similar findings have been presented elsewhere in adolescent psychiatric literature^26,27^.

Considering the above outlined clinical challenges to distinguishing between the described diagnostic concepts, we hypothesize the likely presence of unrecognized pediatric catatonia in peer-reviewed case-studies of pediatric patients diagnosed with PANS. Moreover, we transparently contrast the proposed diagnostic criteria for PANS with catatonia. As briefly outlined above, we argue that PANS and pediatric catatonia can be perceived as diverging disease conceptualizations for youth with severe mental illness and concurrent non-somatic complex motor abnormalities. We hypothesize that the previously inferred under recognition/undertreatment of pediatric catatonia contributes to promoting alternative etiologies/treatments to give an alternative explanation to behavioral anomalies characteristic of catatonia. In effect, could the growing traction of an alternative diagnosis (PANS) partially be explained by an inability to correctly recognize cases of an established psychiatric disorder (pediatric catatonia)? In an exploratory analysis aimed at providing new evidence to add to the current literature on this complex research question, we apply National Registry Data to compare lithium and ECT utilization frequencies across Swedish regions in minors (2016–2020)—regimens that are exclusively recommended for the acute and maintenance treatment of severe mental illness (e.g., psychotic or treatment-resistant severe major depressive disorder, bipolar disorder, schizophrenia or catatonia)^21^, and, thus, plausible surrogate variables to study the extent to which severe mental illness in Swedish minors is recognized and treated according to established guidelines. Lastly, to investigate interrelatedness of scientific literature and whether key differential diagnoses are being overtly considered, we perform bibliographic analyzes of publication networks and abstracted keywords in relation to PANS and pediatric catatonia. We report on results suggesting that (1) catatonic patients are liable to fulfilling diagnostic criteria for PANS, (2) three conservatively assessed PANS case-reports present with possible unrecognized catatonia, (3) lithium and electroconvulsive therapy usage frequencies in Swedish minors (exclusively recommended for severe mental illness) are strongly intercorrelated and unequally distributed across Swedish counties, (4) established severe mental disorders are rarely overtly considered amongst PANS-specific research, (5) there is a rarity of cross-citations across research fields covering PANS and pediatric catatonia despite partly sharing overlapping patients, and (6) best-available evidence treatments appear markedly superior for pediatric catatonia compared to PANS in both childhood and adolescence.

## Methods

### Ethics and selection of PANS-associated case-reports

The case-reports included in this manuscript have been previously published^28,29^. Studies were selectively chosen, underlying the relative scarcity of PANS-associated case-reports. The present study did not include a systematic review of available case-reports and trials, as this has been previously presented^30^. In the initial stage of the article selection process ADB, EJ, JJ, and PA searched for case-reports and case series describing patients diagnosed with PANS. Upon finding a paper describing one or several PANS patients, the individual who found it screened it for inclusion in the consensus in-depth rating. The selection of articles for inclusion for further review at this point was liberal, to avoid missing cases. Thus, cases that the screener was instructed to exclude were those deemed to unequivocally not exhibit signs of catatonia. After an article was included for in-depth consensus review, ADB, EJ, JJ, and PA reviewed the case and scored it according to DSM-5 criteria, the Bush-Francis Catatonia Rating Scale (BFCRS), and the Pediatric Catatonia Rating Scale (PCRS). Ratings at this stage were conducted in a conservative fashion, to minimize bias. Cases where there were disagreements were resolved through consensus discussions. In general, the conservative line was chosen in such discussions. It should however be noted that a systematic review of the entire literature was beyond the scope of this article. Thus, the authors halted the literature search after finding three cases that were deemed appropriate illustrations of signs of pediatric catatonia in case-reports of PANS. All authors later contributed to reviewing the assessments made in the three selected cases.

During this process, a total of 15 individual case-reports were subjected to initial screening (i.e., two^31^ and seven^32^ cases described by Pavone et al., five by Frankovich et al.^28^, and one by Piras et al.^29^, respectively). Five were excluded as part of the screening process—unequivocally deemed not to exhibit catatonia or to present sufficient information to allow for the reasonable distinction of such a diagnosis. Subsequently, ten cases were subjected to in-depth all-author review and consensus discussions—resulting in seven cases considered not representative of catatonia and three cases assessed as exhibiting putative catatonia according to both DSM-5 criteria, BFCRS, and PCRS estimates. (see Supplementary Fig. 1. for an overview of the article selection process).

Of the five cases presented more extensively below, three are the case-reports discussed in the main manuscript. We chose to present two additional cases to illustrate the article selection process. The first of these initial cases were selected as an accurate representation of a case whereby catatonia was clearly not deemed present and where OCD behavior was evidently described as underlying repetitive motor behavior. The second case was chosen since it was deemed an accurate representation of a case where catatonia was possibly present, but where the brevity of the information reported precluded inclusion in the article as an illustrative case of putative pediatric catatonia in a PANS patient.

### Comparison of criteria for PANS and pediatric catatonia

We further developed a table previously published by Benarous et al.^33^, that compares items of catatonia between the DSM-5, the BFCRS, and the PCRS^33^. To investigate the potential overlap of proposed consensus guidelines for PANS suggested by Swedo et al.^4^ and Pfeiffer et al.^5^, we added additional columns that contrast potentially misattributed items of PANS.

### Evaluation of catatonic symptoms in three PANS case-reports

Potential catatonic symptoms described in PANS case reports were transparently assessed by DSM-5 criteria, as well as by BFCRS and PCRS. The BFCRS is a clinician-rated symptom scale designed for adults, but is in clinical practice often implemented in assessments of pediatric catatonia^34^. In screening for catatonia in adults, the BFCRS is considered the gold standard structured instrument^35^. Although the sensitivity is 100%, specificity predictions vary from 75–100% since some of the 23 signs listed are not specific to catatonia. High inter-rater reliability has been demonstrated *r* = 0.93, but due to the waxing and waning nature of catatonia, test-retest reliability has not been demonstrated^36^. In a comparison of six catatonia rating scales, the BFCRS was the most widely used in clinical practice. The BFCRS is regarded as an easy-to-use screening measure for catatonia, with good psychometric properties. Nevertheless, the PCRS, a clinician-rated symptom scale designed to aid in the diagnosis and assessment of symptom severity of catatonia in pediatric populations, has also been proposed. Dhossche et al. emphasizes that catatonia can easily be mistaken for other pediatric syndromes with a similar presentation, such as for instance stereotypies and mutism commonly occurring in ASD^16^. To account for this, additional criteria for diagnosing an episode of pediatric catatonia in populations with developmental disorders are specified in the PCRS—symptoms have to persist for days or weeks and represent a clear deterioration in comparison to the patient´s habitual condition^33^. The PCRS was validated in 2016 in a population of 138 inpatients with (*n* = 88) and without (*n* = 50) catatonia, demonstrating an excellent convergent and discriminative validity for a cutoff value of >8 (AUC = 0.978, sensitivity = 0.95 specificity = 1). Including subjects undergoing treatment with antipsychotics in the control group, conditions more applicable to clinical conditions, psychometric properties remained excellent (AUC = 0.978, sensitivity = 0.97, specificity = 1). When adjusting the cutoff value to 3, specificity remain above 95%, arguably acceptable psychometric properties for traditional research purposes. Selection bias could bias generalizability of the PCRS to outpatient populations, making further validations in these populations desirable^33^. In the DSM-5, catatonia is defined as three or more of the following symptoms: Catalepsy, waxy flexibility, stupor, agitation, mutism, negativism, posturing, mannerisms, stereotypies, grimacing, echolalia and echopraxia^37^. Our evaluation of indications of catatonia according to DSM-5 thus followed this definition.

In order to assess the probability that subjects screened positively for pediatric catatonia truly present with the condition (and vice versa), the positive and negative predictive values were calculated for each respective PCRS score based on visual estimation of sensitivity and specificity values (Benarous et al. did not publish specific values for each cut-off criteria)^33^. Prevalence estimates for pediatric catatonia have previously been reported to vary between 4–17% in different contexts^35,38^. To account for this lack of consensus prevalence estimates, we performed a sensitivity analysis whereby different positive and negative predictive values were calculated based on three different prevalence estimates (4, 10, and 17%) (Supplementary Table 1). Case vignettes for the three cases assessed to demonstrate potential pediatric catatonia and two cases that were not assessed to demonstrate potential pediatric catatonia are provided below.

### Case Viginettes—3 Cases exhibiting potential catatonia

#### Clinical vignette—case 1

In case 1, Frankovich et al.^28^, describe gradual disease onset in a 13-year-old girl, initially marked by the development of extreme anxiety subsequent to initiation of treatment for facial acne with the antibiotic minocycline. The patient was described as a good school performer despite mild cognitive and learning disabilities. Good social functioning and no premorbid psychiatric history was reported at baseline.

The onset of the anxiety is stated to have taken place within a week of initiation of the antibiotic treatment and symptoms went into full remission within 3 days of discontinuation of the medication. However, one month later the patient developed restrictive eating behavior and severe obsessions focused on her dental braces. The onset of these symptoms is reported to have taken place overnight. Nutritional intake is described as a major issue, with the patient having a hard time-consuming food even in cases where ample assistance was provided. A 11-pound weight loss over the 1^st^ week of illness was reported. In addition, insomnia (no sleep for 4 consecutive days), near absence of communication except in regards to the braces, constant use of the right hand to wipe her face, and inconsolable crying and screaming was also reported. Marked functional impairment was also noted, with the patient unable to engage in bathing and other personal hygiene activities. Onset of these symptoms also took place overnight, and they reached their maximum intensity within 24-hours after onset. The symptomatology is reported to have stayed at this intensity with symptoms of continued behavioral regression, preservations, anxiety, cognitive decline, delayed or complete absence of verbal response, repetitive self-soothing behavior, persistent insomnia, poor hygiene, continued weight loss due to low nutritional intake and jaw tremor being reported. Urinary incontinence and extreme urinary frequency appeared three weeks after illness onset. These symptoms eventually necessitated that the patient used a diaper.

After an assessment by a psychiatrist, the patient was diagnosed with bipolar disorder. During the 6 months following that, the condition necessitated inpatient care six times. Psychopharmacological treatments consisting of “medications from nearly every psychotropic medication class” was attempted, with little positive effects reported and notable side effects including sedation, drooling and Parkinsonism. Prescription of benzodiazepines resulted in “severely disinhibited behavior” (sexualized gestures, developmental regression, cognitive impairment). Titration of divalproex, quetiapine, and aripiprazole to full dosages was reported to be without beneficial effects. 1 mg of benztropine twice daily caused sedation but failed to decrease extrapyramidal symptoms. A regimen of lithium and propranolol was reported to have some beneficial mood stabilizing effects, but other psychiatric symptoms continued unabated. Antidepressants and benzodiazepines were also prescribed, and multiple medications were tested to improve sleep, with poor efficacy reported.

Due to the refractory nature of the patient’s symptoms, a referral for ECT was sent 1 year after the initial presentation. A second opinion was also given by a psychiatrist at the Stanford Pediatric Bipolar Disorders program. Due to several features of the condition (sudden onset, unusual course of mania, pronounced OCD symptoms, unsatisfactory response to psychotropic medication, encephalopathic features, persistent tremor, and choreiform movements of fingers), this expert suspected an inflammatory etiology. The patient was thus referred to pediatric neurology and rheumatology departments and evaluations for inflammatory encephalitis and systemic autoimmune diseases were conducted. Following brain imaging, negative infection screens, and serological and cerebrospinal fluid analysis a wide variety of possible organic causes were excluded. However, the blood analysis revealed antineuronal antibodies included in the testing protocol proposed by Dr. Madeleine Cunningham, suggested to be associated with PANDAS and Sydenham’s chorea. Based on these findings, a working diagnosis of inflammatory brain disease, suspected to be striatal encephalitis, informed subsequent treatment. This consisted of initial treatment with high-dose methylprednisolone (1000 mg/daily for 3 days) followed by a slow prednisone taper (60 mg orally twice daily during 4 weeks, thereafter 10% decreased every 3 days). Following the steroid trial, the patient is reported to have returned to 90% of her baseline functioning and psychotropic medications were streamlined. However, attempts to decrease prednisolone below 60 mg daily resulted in re-emergence of symptoms, most notably insomnia and OCD (washing, cleaning, measuring herself). This resulted in re-hospitalization with a second high-dose steroid induction (1000 mg methylprednisolone daily for 3 days) generating what is described as good results. Mycophenylate mofetil was prescribed, to hopefully allow tapering of prednisone without adverse consequences.

During the second attempt to taper prednisone a re-escalation of psychopathology occurred at 15 mg/day, despite the addition of mycophenylate. This re-escalation was described as severe and occurred in conjunction with exposure to Group A streptococcus (GAS) from a close relative (sister), but without the patient, herself testing positive for GAS. Intravenous gamma globulin (IVIG, 2 g/kg) was added monthly for 3 months. Antibiotics to treat and prevent GAS infection were added following positive antineuronal antibody testing with the Cunningham Research Panel.

The patient improved on a treatment course with IVIG and mycophenolate, but a third attempt to taper prednisolone resulted in a third prolonged hospitalization. During these prolonged stays as an inpatient, the patient required a sitter for 24 hours/day, due to the severity of her symptoms (behavior deterioration, OCD symptoms with regards to drinking, including obsessive water drinking, drinking of nail polish remover, and own urine).

When the patient did not respond to steroid escalation during this third hospitalization, plasma exchange therapy (PLEX) (1.5 volume exchange/daily for 3 days) followed by two infusions of rituximab over 2 weeks’ time (750 mg per treatment was administered). This is reported to have resulted in steady improvement and facilitation of successful weaning off prednisone. Functioning of 80% and 100% of baseline was reported at 3- and 6 months post-treatment with no reported psychiatric symptoms including residual OCD, restrictive food intake, anxiety, or insomnia reported. The authors assessed that the patient at this point exhibited “*quiescent disease on aggressive immunosuppressive therapy*”.

After this assessment, the patient is described to have had three disease flares, each of which was reported to be associated with attempts to wean her off immunosuppressive therapy. A minor flare up, consist of behavioral regression, re-emergence of OCD symptoms and polyuria, is reported at 6-month follow-up from initial rituximab/PLEX, assess to coincide with waning therapeutic effects of rituximab and prednisone tapering. This flare up was resolved 1 month after redosing with rituximab. A flare-up described as more severe, encompassing life-threatening impulsivity in addition to the symptoms observed during the first flare-up, was reported to have occurred at 12 month following rituximab/PLEX, also corresponding to waning effects of rituximab and attempts to decrease the dosage of prednisone. A third disease flare up occurred after discontinuation of mycophenolate mofetil, but with adequate treatment with rituximab.

At the conclusion of the case report, the patient is described as having gone through 22 months of relative quiescent disease (functioning at 90% of baseline is reported), with only three disease flares assessed to correspond to the reduction in immunosuppressive therapy and reported as responsive to reinitiating of such therapy. Her new baseline was reported to exclude OCD symptoms, impulsive behavior, sleep dysfunction, anxiety, tremors, and other motoric abnormalities. Testing indicated cognitive function superior to premorbid baseline. At the time of the case report, the patient was living what was described as a normal teenage life at home, after 2 years of living in institutional settings. The patient had started re-attending public school. Concerning pharmacology, the patient was still prescribed mycophenylate, mofetil, rituximab, hyrdroxychloroquine, “low dose”(exact dosage not specified) prednisone, and cefadroxil as GAS prophylaxis. The patient had discontinued psychotropic medication early during the immunotherapy, except for the neuroleptic Quetiapine that was used when needed for sleep and mood stability during her three flare ups. She was reported to function well in school and at home, without the need for any psychotropics.

When asked about prior chorea or OCD the family reported that the patient had abrupt-onset OCD in fourth grade, with a frequent need to urinate, which resolved after 4 days. A friend of hers was also reported to simultaneously have gone through abrupt-onset OCD that lasted for 3 months.

The authors of the case report conclude that they can not conclusively establish that GAS was the trigger for the patient's illness episodes and that it was unknown why the first episode of OCD was relatively mild and self-limited, whilst more pronounced psychopathology was observed during the second episode, necessitating intensive immunotherapy.

#### Clinical vignette case 2

The second case is an 11-year-old boy treated in a US clinic, presenting with sudden onset separation anxiety and rage, 2 weeks after febrile illness with pharyngitis (no throat culture sampled)^28^. Four weeks after the onset of separation anxiety, sudden emergence of OCD symptoms, motoric and vocal tics were observed. OCD symptoms entailed tapping hallway walls, checking and counting rituals, contamination fear, repeating words, a need for symmetry and exactness, and repeating the same question. He had tics consisting of blinking, shoulder and neck movements, and vocal tics (repeating “ga ga ga”). The next month, he exhibited depressed mood, anhedonia, insomnia, and irritability accompanied by violent anger explosions. New physical symptoms in the form of nocturia and severe joint pain (primarily in feet, knees, elbows) necessitating crutches also emerged. Joint pain lasted 3–7 days at a time and co-occurred with escalations in anxiety and rage. Six weeks after onset of psychiatric symptoms, antistreptolysin O and antideoxyribonuclease B titers were taken. Both of these were elevated (368 and 666 Todd units/mL, respectively), assessed as an indication of recent GAS infection. This led to the treating pediatrician suspecting PANDAS. After 5 days of azithromycin, temporary improvement in OCD symptoms was observed (e.g., duration of checking routine prior to bed reportedly decreased from 2 hours to 2 minutes). Anxiety and motoric and vocal tics are said to have completely disappeared. Impaired concentration and impulsivity persisted though, causing continued difficulties in school and scholastic functioning. 5 days following discontinuation of antibiotics, the patient relapsed into psychopathology (anxiety, tics, irritable mood, and OCD). 4 weeks of azithromycin (250 mg/daily) is reported to have conferred quick and stable improvement in psychopathology. 10 days after discontinuation of azithromycin, psychiatric and somatic symptoms re-appeared, prompting the reinstatement of azithromycin with amoxicillin/clavulanate (500 mg/twice per day) added. During follow-up over a year, the patients ASO and anti-DNase B titters fell, but ongoing flare ups of psychiatric and somatic symptoms re-occurred and were assessed to correlate with viral illnesses. After referral to a pediatric immunologist, IVIG (2 g/kg) was prescribed, with the treatment executed 14 months after initial presentation. This treatment was associated with subjective improvement, but the patient suffered an upper respiratory infection 2 weeks after the IVIG treatment. This infection was associated with an acute worsening of the patient’s neuropsychiatric symptoms, that however self-resolved. When the patient was first evaluated in a specialized PANS clinic 22 months after illness onset, he had a pattern of waxing and waning neuropsychiatric symptoms (oppositional behavior, depressed mood, irritability, checking behaviors, motoric tics, pain in joints, heels and neck and nocturia). His symptoms were reported to worsen after viral illnesses, with improvement seen 2–3 weeks later. One more severe exacerbation of symptoms corresponded to increases in AS and anti-DNase B titers. Discontinuation of antibiotics was reportedly attempted many times but, with symptomatology recurring 1–2 weeks after discontinuation. Antibiotics were discontinued circa 2.5 years after onset, and the patient was reported to remain largely free from symptoms with a reported good functional status.

#### Clinical vignette case 3

In case 3, a 7-year-old girl started experiencing recurring episodes of vomiting in the morning for more than one month^29^. Gastrointestinal tests indicated nervous gastritis. The patient is then described to have exhibited early symptoms of OCD (drinking and washing continuously) and sporadic attacks of anger characterized by screaming and cries. Weight gain of more than 10 kg occurred within a few weeks without any dietary changes reported. After this, motor abnormalities of upper and lower limbs and eyes emerged 2 months after the first symptom onset. Whilst no deterioration in scholastic achievement was noted, the quality of the patients handwriting is described as worsened. Repeated medical checks were conducted and stress was assessed as a major contributor to the clinical picture. These pathological signs are described as persisting in moderate intensity for 3 years, after which a sudden increased intensity of OCD, tics, and motor abnormalities were noted, with choreiform finger movements reported to increase in severity. After a consultation with the psychiatric department the patient received an initial diagnosis of Tourette’s syndrome. The patient scored 40 on the Yale Global Tic Severity Scale (YGTSS) and a marked impact on social relationships and daily living was reported. Psychological support, behavioral and medical therapeutics were suggested as interventional methods, however, medication was not prescribed. Following observation of persisting symptoms over a years time prompted a suggestion of PANDAS as a possible diagnosis. Pharmacotherapy with amoxicillin and clavulanic acid were administered during 4 weeks two times per day and substituted with pencillin G benzathine for 18 days and ibuprofen when deemed necessary. This did not improve OCD symptoms, tics, and choreiform movements. Opposing aggressive behavior, depression, psychosis, and auditory hallucinations are described to have emerged at this point. A positive test for Mycoplasma pneumonia prompted administration of clarithromycin with probiotics. After 5 days of this treatment, OCD symptoms were attenuated and the patient exhibited normal walking without compulsions, fear of physical contact disappeared and good mood, handwriting, and learning speed was reported. After 10 days of treatment, further improvement was noted, with almost complete remission of symptoms. An YGTSS score of 18 points were reported at this time. The aggressive attitude and compulsive symptoms are described to have completely disappeared during treatment. Improvement in symptomatology was described as limited to the therapeutic period. Based on metabolic analysis of urine samples collected during the presence of neurological symptoms and after 10 days of treatment, the authors hypothesized that improvements in symptoms were due to the antibiotic treatment.

### Case Viginettes—2 cases not exhibiting potential catatonia

#### Case 1

In this case, a 12-year-old boy presented at an Italian Pediatric Clinic after referral for sudden onset of psychiatric disturbances^31^. The anamnesis included no previous neuropsychiatric abnormalities or movement disorder in the patient or relatives. A previous screening at the patient’s school, resulted in a positive test for SARS-CoV-2, but the child was initially asymptomatic according to the parental report. 2 weeks subsequently, sudden onset of psychiatric symptoms emerged, such as fear of contracting infectious diseases and touching handles, with a strong drive to wash hands very often and “*accurately*”. A reduction in appetite was also noted. Physical examination, including cardiological and neurological assessment evinced no signs of medical illness. Severe emotional lability and facial tics were reported by parents and observed during psychiatric examination. Laboratory tests revealed values in the healthy range and an autoimmunity panel revealed no aberrations. At admission to the psychiatric hospital, a nasal swab testing resulted in a positive test for SARS-CoV-2 2 weeks after the first positive test. EEG, ECG, and brain MRI were normal. The patient scored 22 on the Children’s Yale-Brown Obsessive-Compulsive Scale (CY-BOCS). Psychological treatment was rapidly started. The patient’s anxiety around hand cleanliness and selective eating remained at 2-month follow-up. Motor tics are reported to have remained but are described as “*not constantly present*”. Testing for SARS-CoV-2 was negative at this time point. The mother, however, complained of her child’s irregular writing and lack of attention.

#### Case 2

This case was a 7.5-year-old boy that had suffered from a brief period of motor tics, before exacerbation of psychopathological symptoms that are described as sudden and abrupt^32^. The patient presented with moderate lingual dyskinesia, severe motor tics, and a voice described as an infantile falsetto. No signs of OCD were found. The patient did not respond to azithromycin or neuroleptic medication, but a 1-month full response to corticosteroid treatment was achieved. The authors report that corticosteroid therapy was repeated and that the positive response to this treatment made IVIG treatment superfluous. The patient status was reported to appear normal at an 85-month follow-up.

### Lithium and ECT utilization in Swedish minors (2016–2020)

**Fig. 1:**
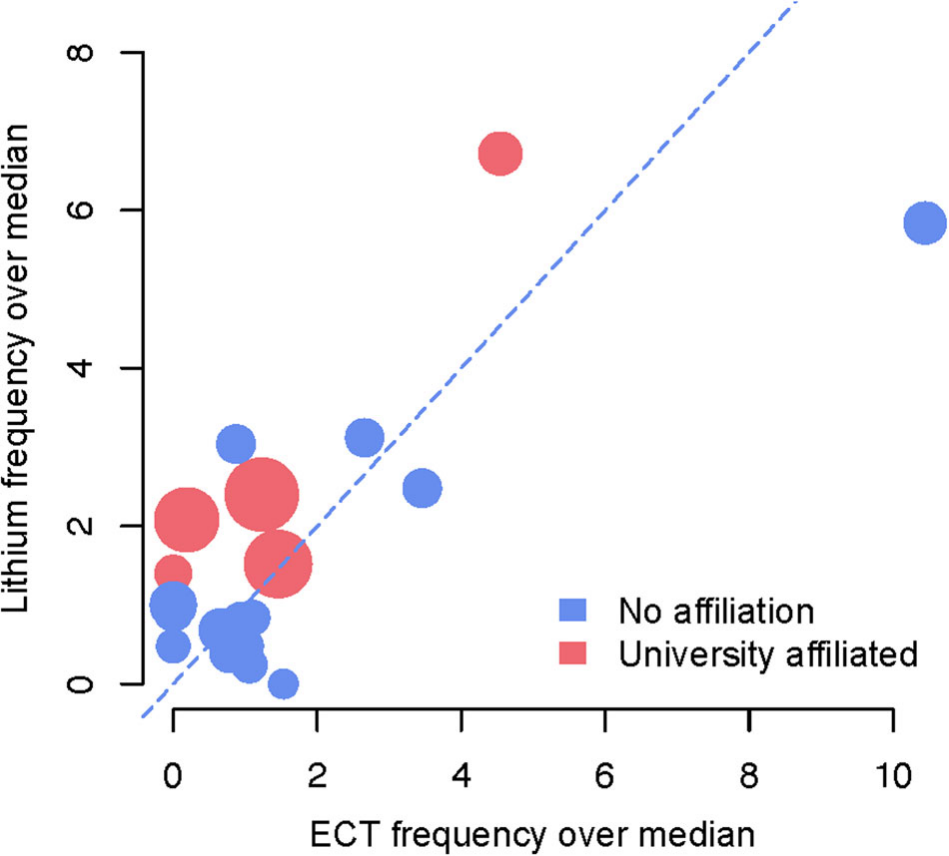
Frequencies of lithium and ECT usage, their deviation from the median, and correlation across Swedish regional councils (2016–2020). Openly available data from the Swedish National Board of Health and Welfare was analyzed to evaluate the frequency of lithium and ECT usage in adolescents for the period 2016–2020, separated by the regional council. For lithium treatment, the number of patients aged 0–17 years and receiving lithium treatment per 1000 inhabitants was extracted for each respective region. Similarly, the number of patients aged 13–17 years and receiving ECT treatment per 1000 inhabitants was extracted from the Swedish ECT registry for each respective region. The figure depicts the usage frequency of lithium and ECT over the national median. University-affiliated counties are highlighted in red, others in blue. County population, retrieved from the Swedish Central Bureau of Statistics, is illustrated by circle diameter. Correlations between ECT and lithium frequencies were assessed by robust linear regression models and the regression slope is illustrated by the dashed line. For a detailed description pertaining to the methods underlying Fig. 1, please see Supplementary Information. Figure 1 demonstrates that (**1**) Five regions in Sweden—caring for 65 736 adolescents and ~11% of Sweden’s population aged 13–17^59^—did not utilize ECT treatment at all in the care of adolescents over a 5-year-period. (**2**) There were large variations in frequencies of advanced treatment usage between regions compared to the national average: 0–6.7× for Lithium and 0–11.1× for ECT. (**3**) Frequencies of ECT and lithium usage were significantly positively correlated across regions (*r* = 0.75, *p* < 0.05). (**4**) There were no significant associations between university-affiliated major regions when compared to other regions in terms of frequencies of ECT (*p* > 0.05) or lithium (*p* > 0.05) usages. (**5**) Lastly, counties affiliated with major cities and university research clinics were not among the five counties with the highest frequencies of ECT treatment usage.

To explore possible geographical variance in the application of advanced psychiatric treatment methods, putatively associated with regional differences in recognition of severe mental illness in pediatric populations, we leveraged ecological data from public databases on the yearly regional usage of two advanced treatment methods exclusively recommended for the treatment of severe mental illness (e.g., lithium and ECT). Openly available data from the Swedish National Board of Health and Welfare and the Swedish ECT registry was analyzed to evaluate the frequency of lithium and ECT usage in adolescents for the period 2016–2020, separated by the regional council (Fig. 1). For lithium treatment, the number of patients aged 0–17 years and receiving lithium treatment per 1000 inhabitants was extracted for each respective region. Similarly, the number of patients aged 13–17 years and receiving ECT treatment per 1000 inhabitants was extracted for each respective region. All statistical analyses were performed using R statistics, version 4.1.0.

Extracted frequencies were non-normally distributed, as measured by the Shapiro-Wilk test (*p* < 0.05). To investigate potential dispersion effects, each region was compared to the national median. Moreover, as data were non-normally distributed, we made use of the ‘lmRob’ function of the “robust” package for R to perform univariate robust linear regressions^39^, studying associations between frequencies of lithium and ECT usage—postulated as plausible surrogate variables to assess the degree of recognition of severe mental illness in children and adolescents. In evaluating the presumption that university hospital-affiliated major regions with populations exceeding one million inhabitants would be associated with higher frequencies of lithium and ECT usage (presumably with superior access to know-how and advanced treatment methods), Wilcoxon signed-rank test was performed by contrasting frequencies with the other regions.

### Bibliography and co-citation in PANS and pediatric catatonia

In brief, published articles pertaining to PANS, PANDAS, and pediatric catatonia were unconditionally retrieved and investigated. Data sets were visualized at an aggregate level in dependency of the relatedness between scientific papers by direct citation (linkage is established between two publications if one cites the other) and by co-occurrences of key search terms (relatedness is determined by the number of articles in which key search terms occur together). In previous attempts to describe different approaches to mapping scientific networks, a framework based on three dimensions has been proposed. The first of these refers to a distinction between social networks, where linkages are established based on the interactions of individuals, and information networks, where relationships between artefacts of science, such as a paper or a journal are the focus of analysis. The second is whether the interrelatedness of two nodes in a network is based on a real connection or an artificial one. The former type of approach includes network-based analysis of co-authorship (formed on the basis of co-authorship of publications) and direct citation-based networks (linkage is established between two publications if one cites the other), the latter co-citation-based networks (e.g., a linkage between two publications is established if they are both cited by a third paper), networks based on bibliographic coupling (e.g., publications are linked on the basis of sharing references), topical networks (e.g., publications or institutions are linked on the basis of the shared topic(s)) and co-word networks (linkages are based on shared words in chosen parts of publications). The third distinction is whether the relationship in focus is based on citation (direct citation, co-citation, bibliographic coupling networks), collaboration (co-authorship networks), or the co-occurrences of words (topical and co-word networks). Yan & Ding propose two dimensions as explaining variance in the networks generated by these methods, one of which is whether the network is based on social relations such as collaboration networks, or cognitive networks based on lexical similarity^40^.

To investigate the relationship between publications on PANS/PANDAS and catatonia, we used a combined approach of two common bibliographical methods; citation analysis and word relationships^41^. In citation analysis, the interrelatedness of academic works is investigated based on their references.

In direct citations relations, which was the method used in the present study, two publications are linked if one cites the other (for a short overview on the topic see, Boyack and Klavans et al.^42^). There has been extensive debate regarding appropriate clustering techniques to adequately represent interlinkages between publications^43,44^. However, recent work has advocated for the use of direct citation analysis to accurately represent the relatedness of publications, with the one caveat that results may be misrepresenting when spanning short time periods^42,45^. As the analysis considered for the present study was not overtly burdened by excessively short time periods for publications, we implemented direct citation analysis for the bibliographic analysis.

In word relationship analysis, shared words in publications are used to indicate interrelationships. This includes words in titles, abstracts, or full text^44,46^. A critique of word analysis is that similar terminology can be used to refer to dissimilar phenomena in different scientific disciplines, and that some general words are commonly used across scientific fields, resulting in the risk that a word analysis approach falsely imputes relationships between fundamentally unrelated publications^45^. As the current analysis is limited to articles within the field of pediatric psychiatry, we anticipated that a relatively well-established and shared nomenclature would alleviate any such concerns and, thus, considered word analysis a relevant approach in this case.

To investigate the information networks of research output on the two topics and overlap/lack thereof, direct citation analysis of documents published on PANS/PANDAS and pediatric catatonia was conducted. Furthermore, to investigate the content of publications and the connections drawn between different concepts, we conducted a co-word analysis of the titles, abstracts, and keywords of our sample of articles.

The methodology used for bibliographical network analysis is described in detail below.

The global literature about PANS and pediatric catatonia was extracted from the online database Web of Science^47^, date 2021–11–07. The following search terms were applied to all searchable fields using one query to identify the closest matching publications: “Pediatric Acute-onset Neuropsychiatric Syndrome”, “Pediatric Autoimmune Neuropsychiatric Disorders Associated with Streptococcal Infections”, “Paediatric Acute-onset Neuropsychiatric Syndrome”, “Paediatric Autoimmune Neuropsychiatric Disorders Associated with Streptococcal Infections”, “PANS/PANDAS”, “pediatric”+“catatonia”, “paediatric”+“catatonia”, “catatonia”+“adolescent”, “catatonia”+“adolescence” or “catatonia”+“childhood”. 568 scientific articles were extracted with full records and cited references. The Vosviewer (previously described in greater detail elsewhere^41,48^) was implemented to perform a direct citation analysis of the extracted data, establishing linkages between scientific papers based on whether or not one cites the other.

In the citation analysis, we focused on the most influential research pertaining to the two topics and specified the minimum number of citations for inclusion of a document to 30. Of the 568 documents, 98 met this threshold. As some of these were not related to each other, only 89 documents in total, the largest set of connected items, were shown in the resulting visualization. In the direct citation visualization, the value for scale was set at 1, item (i.e., document) size was determined by total link strength, coloring of items was chosen according to clusters with the color scheme Viridis and size variation of items was set at 0.5. Size variation for links was set at 1, with a minimum strength of 0 and a maximum strength of 1000. We allowed curved links in our analysis. Normalization was determined by association strength. The settings for “attraction” and “repulsion” in the layout were 2 and 1, respectively. The clustering was done at a resolution of 1 with a minimum cluster size of 1. Small clusters were merged for visualization purposes. We recommend these settings for optimal viewing.

By contrast, for the co-occurrence analysis, we implemented a thesaurus to merge different variants of keywords. All keywords and fractional counting was specified, and the minimum number of occurrences of a keyword was specified to five (which is the default setting). Of 1434 available keywords, 117 met threshold criteria, and were thus included in the subsequent analysis. Normalization was determined by association strength; small clusters were merged, and default layout values were implemented. Size variation and association lines were adjusted for illustration purposes and scale weights were determined by the number of occurrences.

Network visualizations are freely available online:

1. Co-occurrence of keywords: https://app.vosviewer.com/?json=https%3A%2F%2Fdrive.google.com%2Fuc%3Fid%3D1PAazkxxZEZqqors6DUarO9RUeGipc-Tm,
2. Bibliographic direct citation analysis: https://app.vosviewer.com/?json=https://drive.google.com/uc?id=1pinwmebxk0TwfhsVw_reN4ZqoQNXUPa3

### Reporting summary

Further information on research design is available in the Nature Research Reporting Summary linked to this article.

## Results

### Proposed diagnostic criteria for PANS overlap substantially with catatonic items on the DSM-5, BFCRS and PCRS

In Table 1, we demonstrate that unspecific PANS items due to their non-specificity can be attributed to multiple catatonic symptoms. For example, “Sensory or motor abnormalities”—proposed as criteria for PANS—could theoretically also fulfill thirteen catatonic items (i.e., catalepsy, waxy flexibility, posturing, mannerisms, stereotypes, echopraxia, excitement, rigidity, mitgehen, ambitendency, grasp reflex, automatic compulsive movements). Moreover, enuresis is also included in the PCRS and deemed significant for pediatric catatonia^33^. In addition, PANS item “irritability, aggression and/or severely oppositional behaviors” closely mimic catatonic symptoms of agitation, negativism, and combativeness. “Reduced intake of food or fluid”, by Pfeiffer et al. proposed as a definition of severe PANS symptoms, is also included in DSM-5, BFCRS, and PCRS under withdrawal (i.e., refusal to eat, drink and/or make eye contact). Indeed, although not formally included as a diagnostic item, pediatric catatonia has been depicted as a treatable cause of developmental regression^10^, while also included as a PANS item (i.e., “Behavioral (developmental) regression”). There were no evident differences in potential overlap with catatonic symptoms between the guidelines proposed by Swedo et al.^4^ and Pfeiffer et al.^5^.

**Table 1:** Overlapping clinical criteria for catatonia and pediatric acute-onset neuropsychiatric syndrome (PANS).

				PANS			
				(Swedo et al.)*		(Pfeiffer et al.)**	
	DSM-5	BFCRS	PCRS	Criteria	Item	Criteria	Item
Catalepsy (i.e., passive induction of a posture held against gravity)	X	X	X	X	II.6. Sensory or motor abnormalities	X	2.6. Sensory or motor abnormalities
Waxy flexibility (i.e., slight and even resistance to positioning by examiner)	X	X	X	X	II.6. Sensory or motor abnormalities	X	2.6. Sensory or motor abnormalities
Agitation (i.e., not influenced by external stimuli)	X			X	II.3. Irritability, aggression, and/or severely oppositional behaviors	X	2.3. Irritability, aggression, and/or severely oppositional behaviors
Negativism (i.e., opposing or not responding to instructions or external stimuli)	X	X	X	X	II.3. Irritability, aggression, and/or severely oppositional behaviors	X	2.3. Irritability, aggression, and/or severely oppositional behaviors
Posturing (i.e., spontaneous and active maintenance of a posture against gravity)	X	X	X	X	II.6. Sensory or motor abnormalities	X	2.6. Sensory or motor abnormalities
Mannerisms (i.e., odd caricature of normal actions)	X	X	X	X	II.6. Sensory or motor abnormalities	X	2.6. Sensory or motor abnormalities
Stereotypes (i.e., repetitive, abnormally frequent, non-goal directed movements)	X	X	X	X	II.6. Sensory or motor abnormalities	X	2.6. Sensory or motor abnormalities
Echopraxia (i.e., mimicking another’s movements)	X	X	X	X	II.6. Sensory or motor abnormalities	X	2.6. Sensory or motor abnormalities
Excitement (i.e., extreme hyperactivity, constant motor unrest which is apparently non-purposeful. Not to be attributed to akathisia or goal-directed agitation)		X	X	X	II.6. Sensory or motor abnormalities	X	2.6. Sensory or motor abnormalities
Rigidity (i.e., maintenance of a rigid position despite efforts to be moved, exclude if cogwheeling or tremor present)		X	X	X	II.6. Sensory or motor abnormalities	X	2.6. Sensory or motor abnormalities
Withdrawal (i.e., refusal to eat, drink and/or make eye contact)		X	X	X	I. Abrupt, dramatic onset of obsessive-compulsive disorder or severely restricted food intake	X	1. Abrupt, dramatic onset (culmination within 72 h) of … severely restricted food intake.
Impulsivity (i.e., patient suddenly engages in inappropriate behavior without provocation. Afterwards can give no, or only facile explanation)		X		X	II.3. Irritability, aggression, and/or severely oppositional behaviors	X	3. Irritability, aggression, and/or severely oppositional behaviors
Mitgehen (i.e., “anglepoise lamp”, arm raising in response to light pressure or finger, despite instruction to the contrary)		X		X	II.6. Sensory or motor abnormalities	X	6. Sensory or motor abnormalities
Ambitendency (i.e., patient appears motorically “stuck” in indecisive hesitant movement)		X		X	II.6. Sensory or motor abnormalities	X	6. Sensory or motor abnormalities
Gegenhalten (i.e., resistance to passive movement which is proportional to strength of the stimulus, appears automatic rather than willful)		X		X	II.6. Sensory or motor abnormalities	X	6. Sensory or motor abnormalities
Grasp reflex (i.e., per neurological exam)		X		X	II.6. Sensory or motor abnormalities	X	6. Sensory or motor abnormalities
Combativeness (i.e., usually in an undirected manner, with no, or only a facile explanation afterwards)		X		X	II.3. Irritability, aggression, and/or severely oppositional behaviors	X	3. Irritability, aggression, and/or severely oppositional behaviors
Incontinence (i.e., nocturnal enuresis, daytime urinary incontinence, or fecal incontinence)			X	X	II.7. Somatic signs and symptoms, including sleep disturbances, enuresis, or increased urinary frequency.	X	7. Somatic signs and symptoms, including sleep disturbances, enuresis, or increased urinary frequency.
Automatic compulsive movements (i.e., involuntary muscle activity exhibited in posture, attitudes, mimic, or gesture due to inhibition or forced motor action)			X	X	II.6. Sensory or motor abnormalities	X	6. Sensory or motor abnormalities
Schizophasia (i.e., scrambled speech)			X	X	II.4. Behavioral (developmental) regression	X	4. Behavioral (developmental) regression

The first four columns of this table have been previously published by Benarous et al.^14^. Proposed consensus guidelines and clinical criteria for PANS were compared for potential overlap with catatonic symptoms. For visualization purposes, only clinical criteria for catatonia deemed to significantly overlap with PANS were shown. Table 1 demonstrates that unspecific PANS-items due to their non-specificity can be attributed to multiple catatonic symptoms. For example, “Sensory or motor abnormalities” - proposed as criteria for PANS - could theoretically also fulfill catatonic items pertaining to catalepsy, waxy flexibility, posturing, mannerisms, stereotypes, echopraxia, excitement, rigidity, mitgehen, ambitendency, gegenhalten, grasp reflex and automatic compulsive movements. Moreover, enuresis is also included in the PCRS and deemed significant for pediatric catatonia^14^. In addition, PANS item “irritability, aggression and/or severely oppositional behaviours” closely mimic catatonic symptoms of agitation, negativism, and combativeness. “Reduced intake of food or fluid”, by Pfeiffer et al. proposed as a definition of severe PANS-symptoms, is also included in DSM-V, BFCRS and PCRS under withdrawal (i.e., refusal to eat, drink and/or make eye contact). Indeed, although not formally included as a diagnostic item, pediatric catatonia has been depicted as a treatable cause of developmental regression^58^, while also included as a PANS item (i.e., “Behavioural (developmental) regression”).

*Proposed clinical criteria of Pediatric Acute onset Neuropsychiatric Syndrome (PANS) by Swedo et al.^4^.

**Proposed clinical criteria of Pediatric Acute onset Neuropsychiatric Syndrome (PANS) by Pfeiffer et al.^5^.

*PANS* Pediatric Acute onset Neuropsychiatric Syndrome, *DSM-5* Diagnostic and Statistical Manual of mental disorders version V, *BFCRS* Bush-Francis Catatonia Rating Scale, *PCRS* Pediatric Catatonia Rating Scale.

### Three PANS case-reports show convincing signs of catatonia

The first column of this table (criteria…) has been previously published by Benarous et al.^14^. The process of selecting studies is detailed in Supplementary Fig. 1. Putative catatonic cases are referenced below. For transparency, rationale was literally presented in the ‘Rationale’-column, including direct quotes from scrutinized case-reports. Brief rationale was provided, outlining general aspects considered relevant for fulfillment of symptom criteria. Rationale provided for DSM-5 is identical to those presented by Benarous et al.^14^. Descriptions for BFCRS^35^ and PCRS^33^ were literally extracted from the respective assessment instrument. Subjects were conservatively assessed for catatonic symptoms. Putative catatonic symptoms deemed unreliable or too ambiguously descripted, were not included. For full disclosure, please see the complete case-reports referenced below. The presence of pediatric catatonia was deemed probable for subjects simultaneously fulfilling (a) three or more DSM-5 criteria^11^, (b) five or more scored points on the BFCRS^35^, and (c) three or more on the PCRS^33^.

In case 1, a 13-year-old female with mild cognitive impairment is described as presenting with acute-onset symptoms of mutism, stereotypies (“…constant wiping of her face with her right hand”, “repetitive self-soothing”), withdrawal (“She could not feed herself, swallow, or chew, and food would fall out of her mouth if her mother fed her”), impulsivity (“… running into the streets, hiding, and trying to jump out of moving cars”), combativeness (“… constant and obsessive hitting of her parents”) and incontinence (“… she developed urinary incontinence, and extreme persistent urinary frequency of unknown cause, ultimately requiring her to wear diapers”). Notably, a plausible indication of underlying invasive thoughts or ideas was non-existent in the description of the symptoms. By conservative estimates, the patient scored 11 points on the PCRS (Table 2), corresponding to a positive and negative predictive value of 100% and 99–100% (by computed prevalence rates of 4, 10, and 17%), respectively (Supplementary Table 1).

**Table 2:** Conservative assessment of putative catatonic items in published PANS case-reports (Frankovich et al. [2015])—Case 1.

Catatonic criteria	DSM-5	PCRS	BFCRS	Reference (PANS)	Rationale for putative presence of catatonic items
Criteria fulfilled/total score	3	11	13		
Mutism (i.e., no, or very little, verbal response, not applicable if there is established aphasia)	X	2	2	(a) “Nearly absent communication (both talking and writing) except to discuss her braces.” (b) “Her illness stayed at this intensity with continued behavioral regression, cognitive deterioration, anxiety, perseverations, repetitive self-soothing, delayed or absent verbal responses, persistent insomnia, poor hygiene, poor oral intake with ongoing weight loss, and jaw tremor.”	General: No indication of aphasia. DSM-5: Assessment – “no, or very little, verbal response”. PCRS: Assessment – “Speaks less than 20 words/5 min” BFCRS: Assessment – “Speaks less than 20 words/5 min”
Stereotypes (i.e., repetitive, abnormally frequent, non-goal directed movements)	X	3	3	(a) “She also had severe insomnia (not sleeping for 4 consecutive days), nearly absent communication (both talking and writing) except to discuss her braces, constant wiping of her face with her right hand, and inconsolable crying and screaming, and she was unable to engage in daily life activities including bathing and other personal hygiene activities.” (b) “Her illness stayed at this intensity with continued behavioral regression, cognitive deterioration, anxiety, perseverations, repetitive self-soothing, delayed or absent verbal responses, persistent insomnia, poor hygiene, poor oral intake with ongoing weight loss, and jaw tremor.”	General: No indication of invasive thoughts or ideas. Frequency constant. Intensity unclear. DSM-5: Equivalent to “repetitive, abnormally frequent non-goal directed movements”. PCRS: a) Description – “Repetitive, abnormally frequent, non-goal directed movements” b) Assessment – “Constant” BFCRS: a) Description – “Repetitive, non-goal directed motor activity (e.g., finger-play; repeatedly touching, patting or rubbing self); abnormality not inherent in act but in frequency.” b) Assessment – “Constant”
Impulsivity (i.e., patient suddenly engages in inappropriate behavior without provocation. Afterwards can give no, or only facile explanation)	-	-	3	“Her behavioral issues became unmanageable because of her constant and obsessive hitting of her parents, crying and screaming, running into the streets, hiding, and trying to jump out of moving cars.”	General: Item not included in DSM-5 or PCRS. No indication of capacity to subsequently motivate/explain behavior. BFCRS: a) Description – “Patient suddenly engages in inappropriate behavior (e.g., runs down hallway, starts screaming or takes off clothes) without provocation. Afterwards can give no, or only a facile explanation”. b) Assessment – “Constant or not redirectable (impulsivity)”
Withdrawal (i.e., refusal to eat, drink and/or make eye contact)a	X	3	3	(a) “She could not feed herself, swallow, or chew, and food would fall out of her mouth if her mother fed her.” (b) “Her illness stayed at this intensity with continued behavioral regression, cognitive deterioration, anxiety, perseverations, repetitive self-soothing, delayed or absent verbal responses, persistent insomnia, poor hygiene, poor oral intake with ongoing weight loss, and jaw tremor.”	DSM-5: Equivalent to “refusal to eat, drink and/or make eye contact”. PCRS: a) Item – “20. Refusal to eat, drink” b) Description – “Severe decrease of daily food or drink intake” c) Assessment – “No per os intake for one day or more” BFCRS: a) Description – “refusal to eat, drink and/or make eye contect” b) Assessment – “No PO intake/interaction for 1 day or more”
Combativeness (i.e., usually in an undirected manner, with no, or only a facile explanation afterwards)	-	-	2	“Her behavioral issues became unmanageable because of her constant and obsessive hitting of her parents, crying and screaming, running into the streets, hiding, and trying to jump out of moving cars.”	General: Item not present on the DSM-5 or PCRS BFCRS: a) Description – “Usually in an undirected manner, with no, or only a facile explanation afterwards” b) Assessment – “Frequently strikes out, moderate potential for injury”
Incontinence (i.e., nocturnal enuresis, daytime urinary incontinence, or fecal incontinence)b	-	3	-	“Three weeks into the course of this illness she developed urinary incontinence, and extreme persistent urinary frequency of unknown cause, ultimately requiring her to wear diapers.”	General: Item not present on the DSM-5 or BFCRS PCRS: a) Description – “Nocturnal enuresis, daytime urinary incontinence, or fecal incontinence” b) Assessment – “Constant”

Case 1, A 13-year-old female with mild cognitive impairment is described as presenting with acute-onset symptoms of mutism, stereotypies, withdrawal, impulsivity, combativeness, and incontinence^59^. The patient scored 11 points on the PCRS by conservative estimates (Table 2), corresponding to a positive and negative predictive value of 100% and 99–100% (by computed prevalence rates of 4, 10, and 17%), respectively (Supplementary Table 1). For further rationale please see Table 2 and Supplementary Information.

*PANS* Paediatric Acute-onset Neuropsychiatric Syndrome, *DSM-5* Diagnostic and Statistical Manual of mental disorders version V, *BFCRS* Busch-Francis Catatonia Rating Scale, *PCRS* Pediatric Catatonia Rating Scale.

In case 2, an 11-year-old boy with a history of dyslexia and learning disability, presented with acute-onset of negativism (“… oppositional behavior”), stereotypies (“… tapping hallway walls” and “complex vocal tics in which he would repeat ‘Ga ga ga’”), verbigeration (“…repeating words, asking the same question repeatedly”), impulsivity (“… impulsivity”), combativeness (“…rage” and “irritability that was punctuated by violent anger explosions”) and incontinence (“… including nocturia”). Plausible indication of underlying invasive thoughts or ideas was non-existent in the description of the aforementioned symptoms. By conservative estimates, the patient scored 4 points on the PCRS (Table 3), corresponding to a positive and negative predictive value of 58–87% and 100% (by computed prevalence rates of 4, 10, and 17%), respectively (Supplementary Table 1).

**Table 3:** Conservative assessment of putative catatonic items in published PANS case-reports (Frankovich et al. [2015])—Case 2.

Catatonic criteria	DSM-5	PCRS	BFCRS	Reference (PANS)	Rationale for putative presence of catatonic items
Criteria fulfilled/total score	3	4	5		
Negativism (i.e., opposing or not responding to instructions or external stimuli)	X	1	1	“At that time, he had a pattern of waxing and waning neuropsychiatric symptoms (oppositional behavior, irritability, depressed mood, checking behaviors, motor tics) and physical symptoms (joint pains, heel pain, neck pain, and nocturia).”	General: No indication of proportionate contextual triggers, invasive thoughts, or ideas. Frequency unclear. DSM-5: Equivalent to “opposing or not responding to instructions or external stimuli” PCRS: a) Description – “Opposing or not responding to instructions or external stimuli” b) Assessment – “Mild resistance and/or occasionally contrary” BFCRS: a) Description – “Apparently motiveless resistance to instructions or attempts to move/examine patient. Contrary behavior, does exact opposite of instruction” b) Assessment – “Mild resistance and/or occasionally contrary”
Stereotypes (i.e., repetitive, abnormally frequent, non-goal directed movements)	X	1	1	(a) “OCD symptoms included tapping hallway walls, checking rituals, counting rituals, contamination fears, repeating words, asking the same question repeatedly, and a need for symmetry and exactness.” (b) “His tics included blinking, shoulder and neck movements, and complex vocal tics in which he would repeat “Ga ga ga.”“	General: No indication of invasive thoughts or ideas. Frequency & intensity unclear. DSM-5: Equivalent to “repetitive, abnormally frequent non-goal directed movements”. PCRS: a) Description – “Repetitive, abnormally frequent, non-goal directed movements” b) Assessment – “Occasional” BFCRS: a) Description – “Repetitive, non-goal directed motor activity (e.g., finger-play; repeatedly touching, patting or rubbing self); abnormality not inherent in the act but in frequency.” b) Assessment – “Occasional”
Verbigeration (i.e., repetition of phrases or sentences, like a scratched record)	X	2	2	“OCD symptoms included tapping hallway walls, checking rituals, counting rituals, contamination fears, repeating words, asking the same question repeatedly, and a need for symmetry and exactness.”	General: No indication of invasive thoughts or ideas. Frequency unclear – interpreted as repeatedly occurring. Intensity unclear. DSM 5: Equivalent to “repetition of phrases or sentences, like a scratched record”. PCRS: a) Description – “repetition of phrases or sentences, like a scratched record”. b) Assessment – “Frequent”. BFCRS: a) Description – “Repetition of phrases or sentences (like a scratched record”. b) Assessment – “Frequent”
Impulsivity (i.e., the patient suddenly engages in inappropriate behavior without provocation. Afterwards can give no, or only facile explanation)	-	-	1	(a) “His anxiety, motor tics, and vocal tics completely resolved. However, impulsivity and impaired concentration continued, causing difficulties in school and academic functioning.” (b) “The following month he developed a mood disorder characterized by depressed mood, anhedonia, insomnia, and irritability that was punctuated by violent anger explosions.”	General: Item not included in DSM 5 or PCRS. No indication of proportionate contextual triggers, invasive thoughts or ideas. Frequency unclear. BFCRS: a) Description – “Patient suddenly engages in inappropriate behavior (e.g., runs down hallway, starts screaming or takes off clothes) without provocation. Afterwards can give no, or only a facile explanation”. b) Assessment – “Occasional”

Case 2, An 11-year-old boy with a history of dyslexia and learning disability, presented with acute-onset of negativism, stereotypies, verbigeration, combativeness, and incontinence^59^. The patient scored four points on the PCRS by conservative estimates (Table 3), corresponding to a positive and negative predictive value of 58–87% and 100% (by computed prevalence rates of 4, 10, and 17%), respectively (Supplementary Table 1). For further rationale please see Table 3 and Supplementary Information.

*PANS* Paediatric Acute-onset Neuropsychiatric Syndrome, *DSM-5* Diagnostic and Statistical Manual of mental disorders version V, *BFCRS* Busch-Francis Catatonia Rating Scale, *PCRS* Pediatric Catatonia Rating Scale.

In case 3, a 7-year-old girl presented with acute-onset of agitation (“… sporadic attacks of anger with sudden screaming and cries”), negativism (“Strongly opposing aggressiveness and behavior…”), stereotypies (“…like drinking and washing continuously”), combativeness (“… anger with sudden screaming and cries”) and automatic compulsive movements (“… increasingly severe choreiform finger movements”). Plausible indication of underlying invasive thoughts or ideas was non-existent in the description of the aforementioned symptoms^29^. By conservative estimates, the patient scored 5 points on the PCRS (Table 4), corresponding to a positive and negative predictive value of 58–87% and 100% (by computed prevalence rates of 4, 10, and 17%), respectively (Supplementary Table 1).

**Table 4:** Conservative assessment of putative catatonic items in published PANS case-reports (Piras et al. [2020])—Case 3.

Catatonic criteria	DSM-5	PCRS	BFCRS	Reference (PANS)	Rationale for putative presence of catatonic items
Criteria fulfilled/total score	3	5	6		
Agitation (i.e., not influenced by external stimuli)	X	-	-	“She then began to show early symptoms of OCD, like drinking and washing continuously, accompanied by sporadic attacks of anger with sudden screaming and cries.”	General: No indication of triggering external stimuli, invasive thoughts, or ideas. Item not included in the PCRS or BFCRS. DSM 5: Equivalent to “Agitation (i.e., not influenced by external stimuli)”.
Negativism (i.e., opposing or not responding to instructions or external stimuli)	X	3	3	“Strongly opposing aggressiveness and behavior, depression, psychosis, and auditory hallucinations appeared.”	General: No indication of proportionate contextual triggers, invasive thoughts, or ideas. Frequency unclear. Intensity – strong. DSM 5: Equivalent to “opposing or not responding to instructions or external stimuli” PCRS: a) Description – “Opposing or not responding to instructions or external stimuli”. b) Assessment – “Severe resistance and/or continually contrary” BFCRS: a) Description – “Apparently motiveless resistance to instructions or attempts to move/examine patient. Contrary behavior, does the exact opposite of instruction”. b) Assessment – “Severe resistance and/or continually contrary”
Stereotypes (i.e., repetitive, abnormally frequent, non-goal directed movements)	X	2	2	a) “She then began to show early symptoms of OCD, like drinking and washing continuously, accompanied by sporadic attacks of anger with sudden screaming and cries.” b) “The symptoms persisted moderately for about 3 years, which suddenly increased in intensity with OCD, tics, and motor anomalies and increasingly severe choreiform finger movements.”	General: No indication of invasive thoughts or ideas. Frequency unclear. Intensity – severe. DSM 5: Equivalent to “repetitive, abnormally frequent non-goal directed movements”. PCRS: a) Description – “Repetitive, abnormally frequent, non-goal directed movements”. b) Assessment – “Frequent” BFCRS: a) Description – “Repetitive, non-goal directed motor activity (e.g., finger-play; repeatedly touching, patting or rubbing self); abnormality not inherent in the act but in frequency.”. b) Assessment – “Frequent”
Combativeness (i.e., usually in an undirected manner, with no, or only a facile explanation afterwards)	-	-	1	“She then began to show early symptoms of OCD, like drinking and washing continuously, accompanied by sporadic attacks of anger with sudden screaming and cries.”	General: Item not present on the DSM-5 or PCRS. No indication of triggering external stimuli, invasive thoughts or ideas. BFCRS: a) Description – “Usually in an undirected manner, with no, or only a facile explanation afterwards”. b) Assessment – “Occasionally strikes out, moderate potential for injury”

Case 3, A 7-year-old girl presented with acute-onset of agitation, negativism, stereotypies, combativeness, and automatic compulsive movements^31^. The patient scored five points on the PCRS by conservative estimates (Table 4), corresponding to a positive and negative predictive value of 58–87% and 100% (by computed prevalence rates of 4, 10, and 17%), respectively (Supplementary Table 1). For further rationale please see Table 4 and Supplementary Information.

*PANS* Paediatric Acute-onset Neuropsychiatric Syndrome, *DSM-5* Diagnostic and Statistical Manual of mental disorders version V, *BFCRS* Busch-Francis Catatonia Rating Scale, *PCRS* Pediatric Catatonia Rating Scale

### Vast disparities in lithium and ECT usage frequencies across Swedish counties 2016–2020 are strongly correlated and not associated with university affiliation

Five regions in Sweden—caring for 65 736 adolescents and approximately 11% of Sweden’s population aged 13–17—did not utilize ECT treatment at all in the care of adolescents over a 5-year-period. Frequencies of advanced treatment usage varied markedly between regions compared to the national median: 0–6.7× for Lithium and 0–11.1× for ECT (Fig. 1). Frequencies of ECT and lithium usage was strongly positively correlated across regions (*β*_slope_ = 0.75, *p* < 0.05) (Fig. 1). There were no significant associations between university-affiliated major regions when compared to other regions in terms of frequency of ECT (*p* > 0.05) or lithium (*p* > 0.05) usage. Lastly, counties affiliated with major cities and university research clinics were not among the five counties with the highest frequencies of ECT usage (Fig. 1).

### Network visualization indicative of polarized scientific fields in conceptual models for severe mental illness in youth

**Fig. 2:**
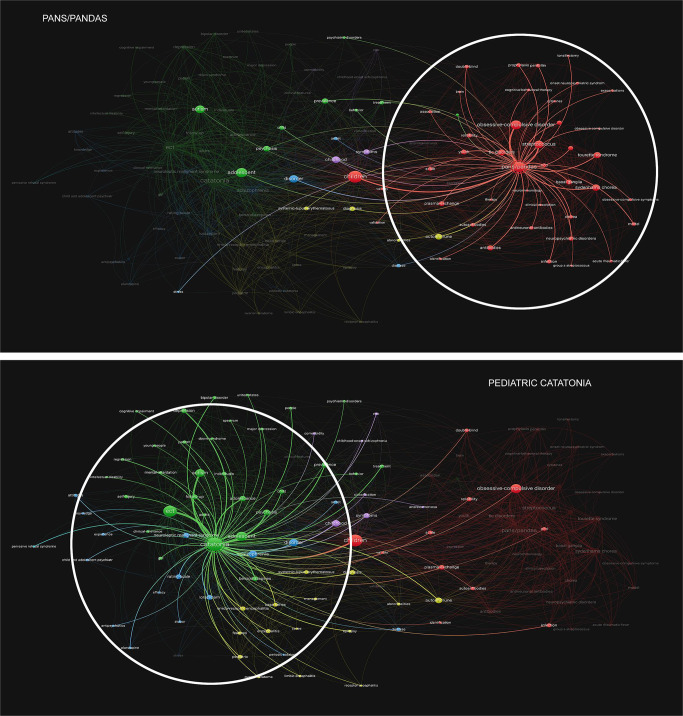
Network visualization of co-occurrence analysis of keywords corresponding to all retrievable articles related to PANS/PANDAS and pediatric catatonia. The Vosviewer software was implemented to unconditionally investigate all available published articles pertaining to PANS, PANDAS, and pediatric catatonia. Bibliographies were extracted from the online Web of Science platform 2021–11–07. Data sets were visualized at an aggregate level in dependency of the relatedness of items determined by the number of articles in which key search terms occur together (co-occurrence of key search terms). For a detailed description pertaining to the methods underlying the bibliographic analysis, please see Supplementary Information. Both figures include the same baseline network visualization. Visual differences pertain to the highlighting of specific keywords and their associated terms. (**1**) Two major clusters were revealed, clearly separating research related to pediatric catatonia from that of PANS and/or PANDAS. (**2**) Research articles including the keyword “catatonia” co-occur with terms pertaining to severe mental disorders, advanced treatments, developmental disorders, and some terms perceived to be associated with immunopsychiatry. (**3**) By contrast, keywords pertaining to PANS and/or PANDAS were associated with infectious agents and complications thereof, neurodevelopmental disorders, medical conditions, autoimmunity, and immunopsychiatry-related treatments. (**4**) Notably, PANS/PANDAS keywords were not associated with any advanced treatment and only “psychosis” among the severe mental disorders.

Two major clusters that clearly separate research related to pediatric catatonia from that of PANS were revealed in the network visualization of the co-occurrence analysis of key search terms. As expected, research articles including the keyword “catatonia” co-occur with terms pertaining to severe mental disorders, advanced treatments, developmental disorders, and some terms perceived to be associated with immunopsychiatry. By contrast, keywords pertaining to PANS and/or PANDAS were mainly associated with probable immunopsychiatric terminology. Notably, PANS and/or PANDAS keywords were not associated with any advanced treatment and only “psychosis” among the severe mental disorders (Fig. 2).

Similarly, two distinct major clusters separating research related to PANS and pediatric catatonia was revealed in the network visualization of the bibliographic direct citation analysis of scientific publications. The field of pediatric catatonia consisted of six sub-clusters, compared with two sub-clusters in the case of PANS/PANDAS, indicating the former as the more diverse field. Relatedness by the number of shared references was nearly non-existent between the two clusters. Studying the two major nodes in each cluster separately revealed no significant across-group relatedness by the number of direct citations.

Network visualizations are freely available online:

1. Co-occurrence of keywords: https://app.vosviewer.com/?json=https%3A%2F%2Fdrive.google.com%2Fuc%3Fid%3D1PAazkxxZEZqqors6DUarO9RUeGipc-Tm,
2. Bibliographic direct citation analysis. https://app.vosviewer.com/?json=https://drive.google.com/uc?id=1pinwmebxk0TwfhsVw_reN4ZqoQNXUPa3

Information detailing settings to improve graphic visualization of the web links are included in the methods section.

## Discussion

Herein, we scrutinized two emerging diagnostic concepts each promoting distinct treatments for youth with acute-onset non-somatic motor abnormalities and severe concurrent psychiatric symptoms (PANS and pediatric catatonia), while also analyzing Swedish National Registry Data to investigate whether severe mental illness is treated equally across counties according to established treatment guidelines in Swedish youth. Previous studies attributed the putative underutilization of advanced treatments in adolescent populations—especially ECT—to service factors, barriers to access, legislative concerns, or lack of training^49^. The findings presented in this study would suggest otherwise. Indeed, we demonstrate that major regions affiliated with prominent research institutions (Karolinska Institute, Gothenburg University, and Lund University)—nationally attributed with state-of-the-art know-how and superior access to advanced treatment methods—did not predict ECT or lithium usage frequencies in Swedish adolescents. Notably, Region Skåne, affiliated with Lund University, hardly utilized ECT treatment at all in the care of adolescents in 2016–2020. Conversely, smaller non-University-affiliated counties accounted for the leading providers. In exploring the putative under-recognition of pediatric catatonia, our results indicate that three peer-reviewed PANS case-reports, conservatively assessed, present with probable catatonia according to DSM-5, BFCRS, and PCRS frameworks—one subject with particularly high probability. As indicated from the re-analyses of PANS case-reports apparently having passed through senior consultants and peer-review processes without acknowledging putative catatonic symptoms, it cannot be completely excluded—and argued to be more likely than unlikely—that the diagnosis received in pediatric patients presenting with acute-onset of severe mental illness and complex non-somatic motor abnormalities without plausible indication of precipitating invasive thoughts or ideas (PANS, pediatric catatonia, or both simultaneously) depend on clinician-related factors (i.e., for example, clinician allegiance or institutional orientation). Bibliographic analyses of literature related to PANS and pediatric catatonia provide further grist to the mill in support of this hypothesis by demonstrating a rarity of cross-citations across research fields despite partly sharing overlapping patients. The overall lack of overt consideration of plausible established differential diagnoses of severe mental illness (appearing more highly prevalent in the PANS field), as indicated by bibliographic analyzes of abstracted keywords, suggest the scope and scale of attitude polarization not to be insignificant. Taken together, these putative controversies could have critical implications for concerned clinicians, who may find themselves perplexed amidst two dominant research fields conferring diametrically opposing directives regarding etiology, nosology, and recommended therapies for minors with acute-onset severe psychiatric symptoms and complex non-somatic motor abnormalities (without plausible indication of precipitating invasive thoughts or ideas). Deducing best-available evidence treatments for minors presenting with such symptoms could, thus, help guide both clinicians and researchers to improve treatment outcomes.

Evidence-based medicine relies on a hierarchy of evidence. Guidelines for severe mental illness in childhood and adolescence predominantly draw conclusions from lower-quality evidence (observational studies and/or extrapolated data from adult populations)^18^. Faced with children and adolescents suffering from severe psychiatric conditions that warrant immediate treatment, clinicians are obliged to inform clinical decision-making on best-available evidence^19^. This conundrum of synthesizing best-practice evidence treatments from a plethora of studies the majority methodologically flawed or of low-quality laden with bias, warrants a balanced perspective^50^. The parachute paradigm informs us of its propriety: despite a complete lack of supporting controlled trials, most will be able to agree on the appropriateness of recommending the use of parachutes when jumping out of airplanes^51^. Leveraging existing data from adult populations may increase the availability of safe and effective pediatric treatments but warrants evaluation of probable risks and benefits of alternative forms of treatment (or of withholding treatments)^52^, and transferability of safety and efficacy. Assessing efficacy in the majority from observational research and extrapolated adult data to inform clinical practice merits investigation of *strength* (size of the observed difference), *consistency* (reproducible treatment outcome results across independent reports confer higher probability of true effectiveness), and *coherence* (evidence supporting a treatment's mechanism of action)^50^. We discuss each of these for pediatric catatonia and PANS, respectively.

*Pediatric catatonia* - there are no established efficacious alternative treatments to lorazepam or ECT^49^. Costs of delaying or withholding treatments are high, i.e., prolonged severe debilitation or potentially death in the case of malignant catatonia^10,14^. Hence, alternative costs to implementing established treatments are high. Furthermore, ECT has been recognized as a safe modality in adolescents by reviews of case-reports, retrospective studies, and overview articles (encompassing several hundred youth). The literature on ECT usage in pre-adolescents is scarce. Yet, existing studies have not provided any indication that safety is a concern in this population^19^. Safety of Lorazepam has been extensively investigated in context of pediatric neurology^53^ and was generally well-tolerated in a prospective naturalistic cohort of 66 children and adolescents with catatonia^22^. In the case of pediatric catatonia, systematic reviews of non-controlled studies report that ECT confer robust response rates of 75% in youth with catatonia^17^, consistent with the more extensive adult response rates of 80–100%^19^. Similarly, Lorazepam had a response rate of 65% in children and adolescents^22^, also conciliable with outcomes in adult populations (70–80%)^54^. The mechanism of action of ECT in catatonia is not yet fully elucidated. However, objective findings in adults support the global effect of ECT in modulating brain structure, connectivity, and function^55^. Moreover, extensive literature supports the action of benzodiazepines on GABAergic and glutamatergic cortical activity, dysregulations of which have been objectively observed (imaging studies) in adult subjects with catatonia^14^. Yet, catatonic features in youth exhibit distinct alterations from the adult clinical picture^14^. In summary, evidence in support of Lorazepam and ECT in the treatment of catatonia in adolescents are overwhelmingly strong when compared to alternative regimens, despite lower-quality evidence in pediatric populations. This information is supported by best-available evidence implicating high alternative costs, well-tolerated safety profiles, and impressive efficacy. While no study implicated serious adverse effects, the relative scarcity of research on ECT in pre-adolescent populations warrant continued caution of its use in this population until fully elucidating safety aspects. Lorazepam, however, constitutes a well-tolerated option with superior evidence for efficacy in the treatment of catatonia in pre-adolescence.

*PANS* - the evidence for the efficacy of treatments for PANS is weak or inconsistent^1^. As outlined above, Lorazepam and/or ECT constitute efficacious alternative treatments to those proposed for PANS (when patients simultaneously fulfill the criteria for pediatric catatonia). Regarding safety profiles of proposed PANS treatments, plasma exchange therapy and intravenous immunoglobins are known to carry substantial risks^8,9^ and a recent systematic review concluded moderate evidence of adverse effects from anti-inflammatory, antibacterial and immunomodulatory treatments in pediatric populations^7^. Efficacy of PANS-related treatments have been inconsistent across reports and evidence synthesis has failed to demonstrate evidence of any beneficial effects^7^. While these studies did not exclusively pertain to subjects with motor abnormalities^7^, no higher-level evidence currently available support the *strength* of PANS-related treatments in these subjects. A recent study by Ferrafiat et al. examined immunomodulatory treatments in subjects with comorbid autoimmune encephalitis (AE; including PANS) and catatonia, postulating catatonic subjects with suspected AE had a good response to treatment. The authors propose a treatment-algorithm including children and adolescents with comorbid catatonia and probable/sero negative AE (i.e., autoimmune condition not substantiated) exempt of treatments for catatonia. Generalizability is, however, prevented in their use of an “exploratory‟ statistical analysis, clinical heterogeneity, center-based bias, recruitment bias, and most importantly from the fact that all catatonic patients received high-dose benzodiazepines implicating that beneficial treatment outcomes could have been conferred by treatments for catatonia^56^. Zheng et al. recently demonstrated that 34 PANS-subjects exhibit distinct imaging findings compared to controls^57^. However, the non-specificity of proposed diagnostic criteria for PANS and putative overlap with other established mental disorders prevent generalizability of such findings. Taken together, hypothesized associations between infectious agents and PANS have not been reliably substantiated by consistent objective findings or reliable biomarkers^6^, resulting in an unsubstantiated (incoherent) pathophysiological model. An understanding of pathophysiology constitutes a basic assumption for any evidence supporting a treatment mechanism of action.

Best-available evidence thus provides strong support for the superiority of established treatments for catatonia in both childhood and adolescence, when compared to treatments for PANS. Our review of selected case-reports indicated probable evidence in support of evident unrecognized catatonia in published PANS case-reports and suggests a significant overlap of diagnostic criteria in patients with complex non-somatic motor abnormalities without plausible indication of precipitating invasive thoughts or ideas. Recent studies depict the preferential implementation of immunomodulatory treatment regimens in minors with comorbid PANS and recognized catatonia, deselecting recommended therapies for catatonia^22,23^. Some studies go so far as to present catatonia as a “red flag‟ for autoimmune conditions advocating early and aggressive immunosuppressive treatments, even without biological evidence in support of such aetiologies^56^. According to the analysis presented in this paper, the clinical implementation of such proposals do not appear to conform with principles of evidence-based medicine. Investigation of the adjunctive value of the addition of immunomodulatory regimens to established treatments for catatonia (i.e., lorazepam, ECT) in pediatric subjects would, however, promote less concern and could be of value to improve existing treatment regimens. Lastly, the increased clinical utilization of structured screening measures (such as the BFCRS or the PCRS) in pediatric populations with motor abnormalities could facilitate increased recognition and treatment of pediatric catatonia.

This study has several limitations. First, due to a retrospective design and variable quality of data available, it cannot be completely excluded that important symptoms and/or clinical assessments were excluded from the reports. Yet, it was possible to conclude that the symptoms identified were simultaneously present. Moreover, the putative presence of catatonia was consistent throughout DSM-5, BFCRS, and PCRS frameworks, providing support for potential diagnosis. It should however be noted that the analyses did not incorporate ICD-11 criteria. Although unlikely, it cannot be ruled out that an analysis based on these might have affected the results. Second, while bibliographic analyses were performed as systematically as deemed reasonable, replication of our findings by independent research groups would be needed to fully elucidate the putative presence of interlinked publication networks and their potential implications. Third, due to data availability issues, the analysis related to the implementation of advanced treatments did not take an international perspective. However, the putative presence of polarized scientific fields, widespread misattribution of probable catatonic symptoms, and the discounting of key differential diagnoses in immunopsychiatric case studies—suggest unequal access to care for minors with severe mental illness may be an issue extending far beyond Sweden’s borders. Fourth, due to data availability issues, the present study did not formally assess the occurrence of any primary psychiatric disorders underlying catatonia. Future studies unconditionally investigating PANS in relation to other severe mental disorders would be valuable. Fifth, the scope and scale of putative misdiagnoses in PANS clinics has not been comprehensively investigated. An independent retrospective chart review of subjects treated at such clinics could further advance our understanding of the extent of diagnostic misconceptions and their real-world consequences. Our study has several strengths. Assessments are transparently presented and reproducible, minimizing risks of bias related to inter-rater reliability and allowing readers to unconditionally scrutinize the validity of evaluations made. Furthermore, in supporting the robustness of our findings, several fundamentally different methods of analyzes based on separate material uniformly implicate lacking severe mental illness prototypes.

This study elicits emerging evidence that partly conflicting conceptualizations of severe mental illness in youth promote unequal access to best-practice care. Incomplete recognition of relevant scientific literature and lack of overt consideration of key differential diagnoses in related fields are plausible contributing factors to the development of these diverging consensuses. National registry data suggest that severe mental disorders in Swedish minors are unequally recognized and treated. Treatment outcomes in a subpopulation of PANS subjects could be markedly improved by prioritizing established treatments for pediatric catatonia in youth with severe mental illness and complex non-somatic motor abnormalities without plausible indication of precipitating invasive thoughts or ideas. Importantly, there are several instances whereby diagnostic criteria were not indicated to overlap, i.e., patients presenting with sensory but not motor abnormalities or patients with complex non-somatic motor abnormalities where there is a plausible indication of precipitating invasive thoughts or ideas.

### Supplementary information

Reporting Summary

Supplementary Information

## Data Availability

Supporting data are available upon reasonable request.
