# Supplementary material for: Youth with severe mental illness and complex non-somatic motor abnormalities: conflicting conceptualizations and unequal treatment
Source: Npj Ment Health Res. 2022 Oct 5;1:13. doi: 10.1038/s44184-022-00013-8 (PMC9533273; doi:10.1038/s44184-022-00013-8)
Supplement: Supplementary file 1 — Reporting Summary [file 44184_2022_13_MOESM1_ESM.pdf]

nature portfolio

Corresponding author(s): Adrian Emmanuel Desai Boström

Last updated by author(s): Aug 23, 2022

Reporting Summary

Nature Portfolio wishes to improve the reproducibility of the work that we publish. This form provides structure for consistency and transparency in reporting. For further information on Nature Portfolio policies, see our Editorial Policies and the Editorial Policy Checklist.

Statistics

For all statistical analyses, confirm that the following items are present in the figure legend, table legend, main text, or Methods section.

n/a	Confirmed	
☒	☐	The exact sample size (*n*) for each experimental group/condition, given as a discrete number and unit of measurement
☒	☐	A statement on whether measurements were taken from distinct samples or whether the same sample was measured repeatedly
☐	☒	The statistical test(s) used AND whether they are one- or two-sided<br>*Only common tests should be described solely by name; describe more complex techniques in the Methods section.*
☐	☒	A description of all covariates tested
☐	☒	A description of any assumptions or corrections, such as tests of normality and adjustment for multiple comparisons
☐	☒	A full description of the statistical parameters including central tendency (e.g. means) or other basic estimates (e.g. regression coefficient) AND variation (e.g. standard deviation) or associated estimates of uncertainty (e.g. confidence intervals)
☐	☒	For null hypothesis testing, the test statistic (e.g. *F*, *t*, *r*) with confidence intervals, effect sizes, degrees of freedom and *P* value noted<br>*Give P values as exact values whenever suitable.*
☒	☐	For Bayesian analysis, information on the choice of priors and Markov chain Monte Carlo settings
☒	☐	For hierarchical and complex designs, identification of the appropriate level for tests and full reporting of outcomes
☒	☐	Estimates of effect sizes (e.g. Cohen's *d*, Pearson's *r*), indicating how they were calculated

Our web collection on statistics for biologists contains articles on many of the points above.

Software and code

Policy information about availability of computer code

Data collection	No software was used in data collection.
Data analysis	All statistical analysis was performed in using R statistics, version 4.1.0.

For manuscripts utilizing custom algorithms or software that are central to the research but not yet described in published literature, software must be made available to editors and reviewers. We strongly encourage code deposition in a community repository (e.g. GitHub). See the Nature Portfolio guidelines for submitting code & software for further information.

Data

Policy information about availability of data

All manuscripts must include a data availability statement. This statement should provide the following information, where applicable:

- Accession codes, unique identifiers, or web links for publicly available datasets
- A description of any restrictions on data availability
- For clinical datasets or third party data, please ensure that the statement adheres to our policy

Supporting data is available upon reasonable request adressed to the corresponding author.

Human research participants

Policy information about studies involving human research participants and Sex and Gender in Research.

Reporting on sex and gender	The study did not involve collection of any original data. Only openly available data was analyzed, and thus we did not assign sex or gender to any of subjects concerned in this data set. The only part of the manuscript where a subjects sex is mentioned is in the case of three previously published case-reports (two girls, one boy), where the authors of the current study have relied on reported sex in the original case reports. Regarding the openly available aggregated data on lithium and ECT usage frequencies, sex specific usage frequencies were not available, and thus not analyzed. Thus, the data utilized in our manuscript did not allow for extensive exploration of the potential role of sex/gender in relationship to the issue of diagnostic challenges in the context of severe mental illness with concurrent motor abnormalities in pediatric populations. However, follow-up studies in this area could adress this important topic.
Population characteristics	The study did not involve collection of any new data and thus, no populations was recruited. The three previously published PANS case reports re-analyzed with the DSM-5 criteria for catatonia, the Bush Francis Catatonia Rating Scale and the Pediatric Catatonia Rating Scale described a 13-year old female with mild cognitive impairment, a 11-year old boy with dyslexia and learning ability and a 7-year old girl, without reported comorbidities, all of which also received a PANS-diagnosis.
Recruitment	No participants were recruited.
Ethics oversight	As the study pertained to openly available data, no ethical permission was required by Swedish jurisdiction. Local university regulatory standards were however followed.

Note that full information on the approval of the study protocol must also be provided in the manuscript.

Field-specific reporting

Please select the one below that is the best fit for your research. If you are not sure, read the appropriate sections before making your selection.

☐ Life sciences ☒ Behavioural & social sciences ☐ Ecological, evolutionary & environmental sciences

For a reference copy of the document with all sections, see nature.com/documents/nr-reporting-summary-flat.pdf

Behavioural & social sciences study design

All studies must disclose on these points even when the disclosure is negative.

Study description	Mixed-methods, qualitative analysis of PANS-criteria and three case reports, quantitative analysis of relationships between ECT and lithium usage frequencies with university affiliation as a covariate and qualitative analysis of bibliographical data.
Research sample	No sample was collected as part of the study. PANS case reports were selectively chosen from the corpus of published case reports on PANS, see Supplementary figure 1. for a detailed description. Analysis of lithium and ECT usage frequencies was done on openly available aggregated data, pertaining to Swedens 21 regions. This dataset is available from the Swedish National Board of Health and Welfare: (https://www.socialstyrelsen.se/en/statistics-and-data/statistics/statistical-databases/)
Sampling strategy	As no new data was collected as part of the study, no sample was drawn from any population.
Data collection	No new data was collected as part of the study.
Timing	Not applicable. However, the analysis of the openly available data described in the manuscript was conducted beteween 2021-09-01 and 2022-01-10.
Data exclusions	No data was excluded from analysis.
Non-participation	Not applicable, as no new data was collected.
Randomization	Not applicable, as no new data was collected.

Reporting for specific materials, systems and methods

We require information from authors about some types of materials, experimental systems and methods used in many studies. Here, indicate whether each material, system or method listed is relevant to your study. If you are not sure if a list item applies to your research, read the appropriate section before selecting a response.

Materials & experimental systems

n/a	Involved in the study
☒	☐ Antibodies
☒	☐ Eukaryotic cell lines
☒	☐ Palaeontology and archaeology
☒	☐ Animals and other organisms
☒	☐ Clinical data
☒	☐ Dual use research of concern

Methods

n/a	Involved in the study
☒	☐ ChIP-seq
☒	☐ Flow cytometry
☒	☐ MRI-based neuroimaging
