# Supplementary material for: Youth with severe mental illness and complex non-somatic motor abnormalities: conflicting conceptualizations and unequal treatment
Source: Npj Ment Health Res. 2022 Oct 5;1:13. doi: 10.1038/s44184-022-00013-8 (PMC9533273; doi:10.1038/s44184-022-00013-8)
Supplement: Supplementary file 2 — Supplementary Information [file 44184_2022_13_MOESM2_ESM.docx]

**Supplementary notes**

**The Bush-Francis catatonia rating scale (BFCRS), the pediatric catatonia rating scale (PCRS) and established DSM-5 criteria for catatonia**

The Bush-Francis Catatonia Rating Scale (BFCRS) is a clinician rated symptom scale designed for adults, but is in clinical practice often used to rate prevalence and severity of pediatric catatonia1. The Bush-Francis Catatonia Rating Scale is considered the gold standard for catatonia screening^2^. The sensitivity is 100%, but specificity predictions range from 75-100% as some of the 23 signs are not specific to catatonia. The measure has a high inter-rater reliability (r=0.93 correlation coefficient), but test-retest reliability has not been proven as the natural course of catatonia waxes and wanes^3^. When compared against five other catatonia rating scales, the Bush-Francis Catatonia Rating Scale was the most widely used. It is regarded as a valid, reliable, and user-friendly catatonia rating scale. Nevertheless, the Pediatric Catatonia Rating Scale (PCRS), a clinician rated symptom scale designed to aid in the diagnosis and assessment of symptom severity of catatonia in pediatric populations, has also been proposed. Dhossche et al. emphasizes that catatonia can easily be mistaken for other pediatric syndromes with similar presentation, such as for instance stereotypies and mutism commonly occurring in ASD^4^. To account for this, additional criteria for diagnosing an episode of pediatric catatonia in populations with developmental disorders are specified in the PCRS – symptoms have to persist for days or weeks and represent a clear deterioration in comparison to the patient´s habitual condition^5^. The PCRS was validated in 2016 in a population of 138 inpatients with (n=88) and without (n=50) catatonia, demonstrating an excellent convergent and discriminative validity for a cut-off value of >8 (AUC = .978, sensitivity = .95 specificity = 1). Including subjects undergoing treatment with antipsychotics in the control group, conditions more applicable to clinical conditions, psychometric properties remained excellent (AUC=.978, sensitivity= .97, specificity = 1). When adjusting the cut-off value to 3, specificity remain above 95 %, arguably acceptable psychometric properties for traditional research purposes. Selection bias could bias generalizability of the PCRS to outpatient populations, making further validations in these populations desirable^5^. In the DSM-5, catatonia is defined as three or more of the following symptoms: Catalepsy, waxy felxibility, stupor, agitation, mutism, negativism, posturing, mannerisms, stereotypies, grimacing, echolalia and echopraxia^6^. Our evaluation of indications of catatonia according to DSM-5 thus followed this definition.

**References**

1 Ridgeway L, Okoye A, McClelland I, Dhossche D, Kutay D, Loureiro M. Case Report: A Case of Pediatric Catatonia: Role of the Lorazepam Challenge Test. *Front Psychiatry* 2021; **12**: 637886.

2 Bush G, Fink M, Petrides G, Dowling F, Francis A. Catatonia. I. Rating scale and standardized examination. *Acta Psychiatr Scand* 1996; **93**: 129–136.

3 Sienaert P, Rooseleer J, De Fruyt J. Measuring catatonia: a systematic review of rating scales. *J Affect Disord* 2011; **135**: 1–9.

4 Dhossche DM, Wachtel LE. Catatonia is Hidden in Plain Sight Among Different Pediatric Disorders: A Review Article. *Pediatr Neurol* 2010; **43**: 307–315.

5 Benarous X, Consoli A, Raffin M, Bodeau N, Giannitelli M, Cohen D *et al.* Validation of the Pediatric Catatonia Rating Scale (PCRS). *Schizophr Res* 2016; **176**: 378–386.

6 Walther S, Strik W. Catatonia. *CNS Spectr* 2016; **21**: 341–348.

| **Supplementary Table 1. Sensitivity table measuring positive and negative predictive values for different cut-off values for the Pediatric Catatonia Rating Scale** | | | | | | | | | | |
| --- | --- | --- | --- | --- | --- | --- | --- | --- | --- | --- |
|  |  |  |  | **Prevalence estimate** | | |  | **Prevalence estimate** | | |
|  |  |  |  | **4%** | **10%** | **17%** |  | **4%** | **10%** | **17%** |
|  |  |  |  |  |  |  |  |  |  |  |
| **Score >** | **Sensitivity** | **Specificity** |  | **PPV** | **PPV** | **PPV** |  | **NPV** | **NPV** | **NPV** |
| 1 | 100% | 85% |  | 22% | 43% | 58% |  | 100% | 100% | 100% |
| 2 | 98% | 89% |  | 27% | 50% | 65% |  | 100% | 100% | 100% |
| 3 | 98% | 96% |  | 51% | 73% | 83% |  | 100% | 100% | 100% |
| 4 | 98% | 97% |  | 58% | 78% | 87% |  | 100% | 100% | 100% |
| 5 | 98% | 97% |  | 58% | 78% | 87% |  | 100% | 100% | 100% |
| 6 | 98% | 98% |  | 67% | 84% | 91% |  | 100% | 100% | 100% |
| 7 | 98% | 98% |  | 67% | 84% | 91% |  | 100% | 100% | 100% |
| 8 | 96% | 99% |  | 80% | 91% | 95% |  | 100% | 100% | 99% |
| 9 | 94% | 100% |  | 100% | 100% | 100% |  | 100% | 99% | 99% |
| 10 | 93% | 100% |  | 100% | 100% | 100% |  | 100% | 99% | 99% |
| 11 | 93% | 100% |  | 100% | 100% | 100% |  | 100% | 99% | 99% |
| 12 | 88% | 100% |  | 100% | 100% | 100% |  | 100% | 99% | 98% |
| 13 | 83% | 100% |  | 100% | 100% | 100% |  | 99% | 98% | 97% |
| 14 | 81% | 100% |  | 100% | 100% | 100% |  | 99% | 98% | 96% |
| 15 | 78% | 100% |  | 100% | 100% | 100% |  | 99% | 98% | 96% |
| 16 | 72% | 100% |  | 100% | 100% | 100% |  | 99% | 97% | 95% |
| **Legend (Supplementary Table)**: In order to assess the positive and negative predictive values from derived PCRS-scores, we estimated cut-off specific sensitivity and specificity values using visual estimates from an ROC curve (Benarous *et al*. did not publish specific values for each cut-off criteria)^1^. Prevalence estimates for pediatric catatonia has previously reported to vary between 4-17% in different contexts. To account for this the lack of consensus prevalence estimates, we performed a sensitivity analysis whereby different PPV and PPV values were calculated based on three different prevalence estimates (4, 10 and 17%). Abbreviations: NPV, negative predictive value; PPV, positive predictive value.  **References (Supplementary Table)** **1.** Benarous X, Consoli A, Raffin M, Bodeau N, Giannitelli M, Cohen D *et al.* Validation of the Pediatric Catatonia Rating Scale (PCRS). *Schizophr Res* 2016; **176**: 378–386. | | | | | | | | | | |

**Supplementary Figure 1. Flow-chart depicting the article selection process**


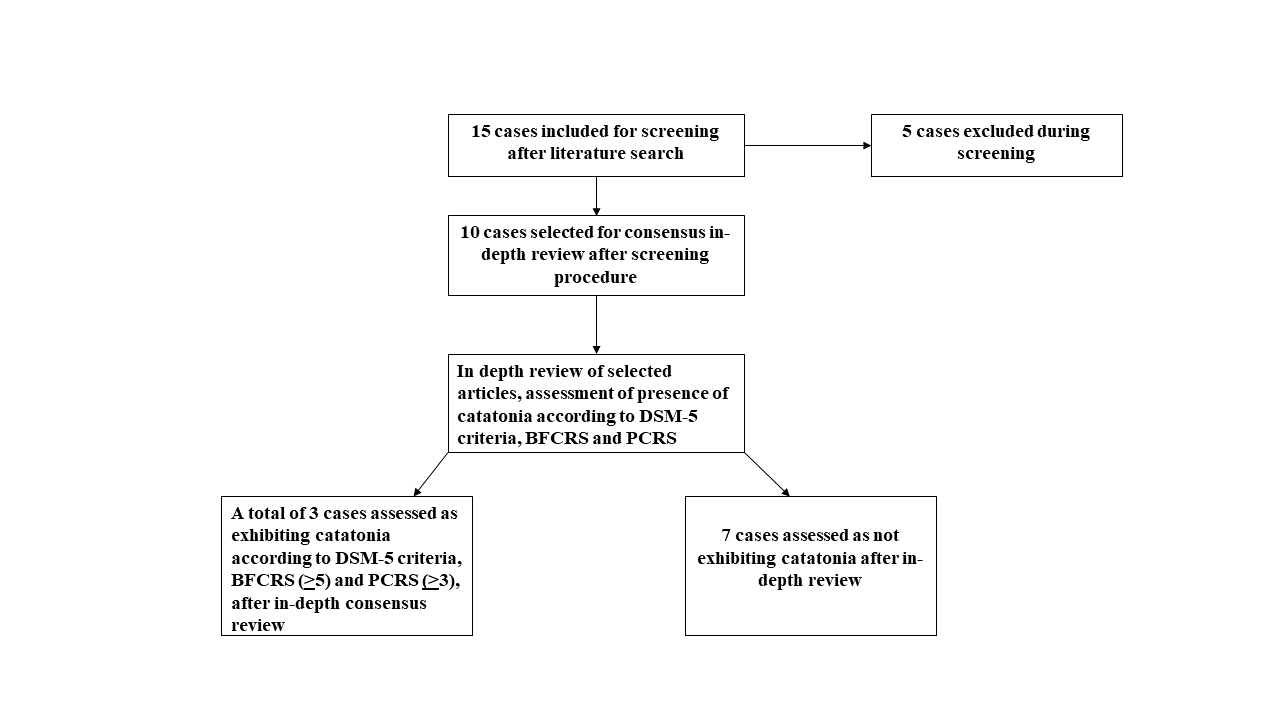


**Legend (Supplementary Figure 1.):** During this process, a total of 15 individual case-reports were subjected to initial screening (i.e., two^1^ and seven^2^ cases described by Pavone et al., five by Frankovich et al.^3^ and one by Piras et al.^4^, respectively). Five were excluded as part of the screening process – unequivocally deemed not to exhibit catatonia or to present sufficient information to allow for the reasonable distinction of such a diagnosis. Subsequently, ten cases were subjected to in-depth all-author review and consensus discussions – resulting in seven cases considered not representative of catatonia and three cases assessed as exhibiting putative catatonia according to both DSM-5 criteria, BFCRS and PCRS estimates. For a detailed description of the article selection process, please see **Supplementary Notes.**

**Supplementary Figure References**

1. Pavone, P. *et al.* SARS-CoV-2 related paediatric acute-onset neuropsychiatric syndrome. *Lancet. Child Adolesc. Heal.* **5**, e19 (2021).

2. Pavone, P. *et al.* Severe Psychotic Symptoms in Youth with PANS/PANDAS: Case-Series. *J. Child Adolesc. Psychopharmacol.* **30**, 567–571 (2020).

3. Frankovich, J., Thienemann, M., Rana, S. & Chang, K. Five Youth with Pediatric Acute-Onset Neuropsychiatric Syndrome of Differing Etiologies. *J. Child Adolesc. Psychopharmacol.* **25**, 31 (2015).

4. Piras, C. *et al.* Pediatric Acute-onset Neuropsychiatric Syndrome and Mycoplasma Pneumoniae Infection: A Case Report Analysis with a Metabolomics Approach. *Curr. Pediatr. Rev.* **16**, 183 (2020).
